# A Manifesto for Universal Healthcare: Reconstituting Primary Care Through Digital Innovation, Microbial Technologies and Empowered Citizenship

**DOI:** 10.1111/1751-7915.70345

**Published:** 2026-04-20

**Authors:** Kenneth Timmis, Gerard Clarke, María Francisca Colom, Zeynep Ceren Karahan, Rachel Armstrong

**Affiliations:** ^1^ Institute for Microbiology Technical University of Braunschweig Braunschweig Germany; ^2^ APC Microbiome Ireland, Department of Psychiatry and Neurobehavioral Sciences University College Cork Cork Ireland; ^3^ Department of Plant Production and Microbiology, San Juan de Alicante, Institute for Sanitary and Biomedical Research of Alicante Miguel Hernández University Alicante Spain; ^4^ Department of Medical Microbiology, School of Medicine Ankara University Ankara Turkey; ^5^ Department of Architecture KU Leuven Gent Belgium

## Abstract

Despite unprecedented medical advances, global healthcare systems are failing to deliver universal, equitable and quality care. Many systems also have low resilience to surges in demand, are highly fragmented, or suffer from unsustainable funding models. The crisis of poor accessibility of healthcare services, which includes both lack of availability and unacceptably long waiting times, stems from systemic failures: inadequate provision of primary healthcare, suboptimal deployment of human and non‐human healthcare assets, metric‐ and profit‐centric models that exacerbate inequality, fragmented and siloed services, unsustainable costs and a reactive focus on treatment over prevention. Climate change and demographic shifts threaten to overwhelm already strained systems. In this discourse we argue that achieving the fundamental human right to healthcare requires a radical reconstitution of primary healthcare, centred on unlocking previously un‐ and under‐exploited resources, capacities and productivity, governed by the principle of ‘Networked Agency with a Safety Net’. We propose a holistic transformation that increases accessibility, resilience, integration, sustainability and, crucially, equity, centred on three synergistic pillars. First, a digital and patient‐agency revolution, designed to radically increase access to, and the productivity of, primary healthcare. This involves creating self‐care ecosystems such as Do‐It‐Yourself Digital Medical Centres and Home Clinics, supported by a National Clinical Informatics Centre. By enabling patients to manage routine care, this system frees highly trained healthcare professionals to focus on the complex clinical work that demands their full expertise. This enables and fosters patient empowerment while ensuring continuous clinical oversight (*to prevent any misunderstanding: clinical oversight means that all clinical recommendations/decisions are made by healthcare professionals; patient agency involves inter alia implementing such recommendations/decisions*). Second, acceleration of the strategic exploitation of microbial technologies—frugal, sustainable tools for diagnostics, prophylactics and therapies, including and especially mental health interventions, and environmental health (One Health). Third, a decisive shift towards disease prevention and health creation, integrating ‘Health in All Policies’, targeted comprehensive health education, and a comprehensive and systematic dismantling of healthcare accessibility barriers—such as transport impediments—and legacy forms of discrimination like restricted sexual/reproductive healthcare and failure to adequately care for the most chronically underserved, including the ageing population. This model is inherently sustainable and designed to drastically reduce the healthcare sector's carbon footprint and environmental impact through service consolidation, transport‐oriented siting and green infrastructure. The measures constitute a technical upgrade and also a fundamental recasting of the primary healthcare system and mindset. This is also a moral imperative. Governments, while increasingly delegating service provision to commercial actors, hold a non‐delegable *duty of care*. Fulfilling this duty necessitates a covenant that transitions healthcare from a market commodity to a publicly‐accountable system sustainably designed for long‐term resilience, equity and dignity. The roadmap we provide—encompassing governance, infrastructure, innovation and education—charts a course from crisis to a sustainable future where universal access to quality healthcare can finally be realised.

## Graphical abstract

Despite unprecedented medical advances, global healthcare is failing to deliver universal, equitable and quality care, vulnerable to surges in demand, and unsustainable. Here we describe a self‐reinforcing framework for rebuilding equitable and resilient health systems. It consists of four integrated domains—patient and professional agency, an intelligent clinical informatics loop, rationalisation of physical and digital infrastructure, and redirection of innovation toward equity—that create a continuous cycle of enablement and feedback. The cycle is framed and governed by a Global Healthcare Access Covenant that catalyses and safeguards systemic transformation.

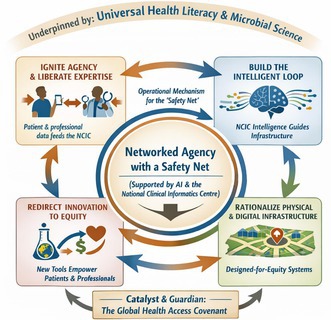

## 1 The Principle of ‘Healthcare is a Human Right’

Poor health can impact every aspect of human endeavour and life experience, most directly in terms of personal suffering but more broadly in terms of personal advancement through education and career, fulfilment of personal potential and aspirations, interactions with others, including family‐friend social and support networks and the burdens these networks may bear. It is, for many, together with happiness and love, the most important aspect of life. The consequences of poor health for individuals, communities and nations are huge.

International organisations, the governments of their member states, and many individuals hold that healthcare is a human right: ‘the enjoyment of the highest attainable standard of health is one of the fundamental rights of every human being without distinction of race, religion, political belief, economic or social condition’ (1946 Constitution of the World Health Organization; United Nations 2008). Key elements of this right are *availability* of ‘functioning health facilities, goods and services for all’ and *accessibility*: ‘non‐discrimination, physical accessibility, economic accessibility (affordability) and information accessibility’.

The Sustainable Development Goals (SDGs) formulated by the United Nations (2015) constitute an influential roadmap designed to channel and maximise progress towards a more sustainable future. The ambitious overarching goal is encapsulated in the powerful and inspiring phrase ‘leaving no‐one behind’. SDG 3—*Ensure healthy lives and promote well‐being for all at all ages*—focuses on global human health. It consists of 9 rather specific sub‐goals plus 4 more general ones. Subgoal SDG 3.8 *Achieve universal health coverage, including financial risk protection, access to quality essential health‐care services and access to safe, effective, quality and affordable essential medicines and vaccines for all* is, however, generic and arguably the most important and overarching ambition. It is the challenge of universal access to quality healthcare services that is the subject of this Editorial.

‘Universal health coverage means that all people have access to the full range of quality health services they need, when and where they need them, without financial hardship. It covers the full continuum of essential health services, from health promotion to prevention, protection, treatment, rehabilitation and palliative care across the life course’ (Knaul et al. 2018; World Health Organization 2025b). This definition prescribes not only the quality and range of services but also parameters of

- time, that is, how long a patient must wait for a healthcare service—‘waiting time’,
- place: the logistical issue of bringing together patient, clinician and diagnostic‐therapy infrastructure, which can be challenging for many healthcare systems and the patients they serve, particularly those in under‐resourced and rural settings, and
- cost: provision of a healthcare service should not engender financial hardship.

Providing quality healthcare to its population is one of the primordial responsibilities of every government, frequently articulated in ‘charters’ (e.g., https://www.alberta.ca/alberta‐health‐charter; Frenk and Moon 2023). Effective confrontation of the challenges presented by SDG 3.8 is thus a global political, economic, humanitarian, moral and ethical imperative (Lomazzi 2016; American Public Health Association 2025).

## 2 The Problem: The Practice of ‘Healthcare is a Human Right’

### 2.1 Cost Issues Limit Access and Entrain Inequalities

While healthcare may be a human right, its huge financial cost challenges the principle of universal health coverage (UHC) (World Health Organisation 2025a). Political decisions on healthcare are, understandably, often based primarily on immediate economic considerations, often overlooking wider ramifications of ill health including

- burdens on the family‐friend network/carers, including any financial burdens incurred, potential impacts on education‐career‐personal fulfilment of the various actors involved, and consequences for community‐regional‐national economic prosperity, and
- the physical and mental suffering of the patient, a parameter which is to some extent but not entirely captured in the term Quality‐Adjusted Life Years (QALYs).

It is essential that health policy formulation and implementation give greater consideration to the collateral humanitarian effects of ill‐health.

In spite of exhortations for a ‘grand convergence of healthcare provision within a generation’ (e.g., Jamison et al. 2013), and the view that ‘Investing in health is one of the most powerful drivers of economic growth and job creation’ (Watkins et al. 2018; World Bank 2025), in a time of unprecedented medical advances, global healthcare systems are struggling to provide services, sometimes even basic services, to their populations (e.g., Darzi 2024). According to the *Tracking universal health coverage 2025 global monitoring report* ‘As of 2023, an estimated 4.6 billion people still lacked coverage for essential health services, and in 2022, 2.1 billion people experienced financial hardship due to out‐of‐pocket health spending’ (World Bank 2025). Insufficient healthcare access represents a major and amplifying element of societal inequality (World Health Organisation 2023a).

Importantly, biomedical research is continually discovering new ways of diagnosing, preventing and treating health conditions, but the translation of such discoveries into clinical practice is often expensive and a further cause of rising costs. This commercial dynamic not only makes existing solutions unaffordable but also fundamentally skews the pipeline of new discoveries away from the health conditions that represent the greatest global burden of disease, particularly in Low‐ and Middle‐Income Countries (LMICs). As a result, solutions to clinical problems abound but many cannot be afforded, or cannot be afforded in under‐resourced settings.

Demographic changes are amplifying rising healthcare costs because, although people are increasingly living longer, in most settings life extensions are not yet health extensions, so the number of poor health life years increases with increases in life expectancy (Crimmins 2015; Coe et al. 2022; Nemitz 2022; Garmany and Terzic 2024; Mather and Revelas 2025). Thus, while extending life expectancy is generally considered to reflect the success of healthcare, the burden it generates increasingly prejudices the ability of healthcare services to deliver.

In some countries, healthcare is financed primarily from the public purse, in others through private insurance and, in the majority, through a combination of public and private systems. While publicly‐funded healthcare has the potential for social equality, systems with private, usually tiered (increasing premiums cover increasing services) insurance almost by definition have unequal provision of healthcare with those unable to afford private insurance, or at least adequate insurance, being significantly disadvantaged (‘Insurance is not just supposed to get you access to care, it's supposed to keep you from getting evicted from your apartment because you paid your hospital bill instead of your rent’.; Powell 2016).

### 2.2 Impact of the Financialisation, Corporatisation and Commodification of Healthcare on Its Accessibility and Quality

Currently, there is a worrying trend of increasing transition of public healthcare services into private ownership (European Network of Corporate Observatories 2021; Marchandot and Morel 2025; Suzuki et al. 2025). The justification for this is the notion of increased efficiency and professionalism of service delivery. However, since the driving force of business is profit generation, costs are preordained to increase, since there is little or no incentive for increasing efficiency and cost reduction, despite documented wastage of resources (OECD 2017). Indeed there is evidence that public health services may be more cost effective than privatised services, ‘despite public hospitals operating in far greater numbers in rural and regional areas—traditionally much more expensive to service—and despite their high‐cost responsibility for providing accident and emergency room services’ (The McKell Institute 2014), so the validity of the justification for privatisation may be questionable.

Several studies suggest that privatisation of healthcare facilities is associated with a decline in the quality of services and poorer health outcomes (Duggan et al. 2023; Keene 2023; Goodair and Reeves 2024; Plourde 2025). Importantly, privatisation can also exclude significant proportions of the population from quality healthcare, in some cases even basic healthcare, because of insufficient or lack of private insurance (https://siepr.stanford.edu/news/study‐when‐public‐hospitals‐go‐private‐low‐income‐patients‐lose). Privatisation may also fragment unified national health services, reducing cohesion, responsiveness, system resilience and ability to cope with national imperatives and surges in need (Tansey 2021). **Crucially, privatisation transmutes a political obligation of universal quality healthcare into an economic obligation of profit generation, with concurrent obfuscation of elemental human rights and governance responsibilities**.

### 2.3 Systemic Discrimination in Healthcare Engendered by a ‘One‐Size‐Fits‐All’ Mindset and the Need for People Centred Care

#### 2.3.1 The Rise in Metric‐Driven Healthcare

In step with the trend to transfer healthcare services into the private sector, there has been growth in both private and public healthcare in ‘managerialism’, the ‘professionalisation’ of healthcare service provision by managers—experts in administration and organisation—in some cases at the expense of healthcare professionals. Healthcare is increasingly run along business lines driven by profit targets and other commercial metrics, with healthcare professionals, who are the major stakeholders in healthcare provision, having a decreasing input into policy. Quite apart from any practices imposed by managers that may disadvantage patients, there is also the issue that they can and sometimes do create bureaucracies that are top‐heavy—not reflecting system needs—and costly (see OECD 2017; Yang and Grenier 2024). In times of flat or marginally increasing healthcare budgets, this imbalance may limit and even reduce the number and quality of frontline healthcare professionals and the services they provide.

The diversity of patient genetics, physiologies, lifestyles, nutrition and environmental contexts, the diversity of diseases—physical and mental—that can affect them, combined with the diversity of microbiome compositions and their impacts on health and disease, are increasingly recognised as factors determining complex ecophysiological landscapes for some diseases that engender individual treatment needs: the practice of precision/personalised medicine. While high‐end healthcare has embraced precision medicine as either a transformative advance or lucrative activity, it is not accessible to the majority of patients, nor is it likely to become so in the near future.

Moreover, the allocation of defined short time slots for consultations ignores vital needs for informative patient:clinician dialogue for exploration of effective diagnosis‐treatment pathway space and all‐important relationship confidence building. There is a world of difference between acquisition of essential information from a carpenter presenting with a cut finger and an old person with multimorbidities in cognitive decline presenting with abdominal discomfort or chronic tiredness. Consultation times (and other services) must accommodate the patient and issue at stake, not the other way round (Irving et al. 2017).

The perception that healthcare services can be programmed to fit standard metrics and targets—the notion of one‐size‐fits‐all—runs counter to the recognition of disease, patient and treatment granularity. It not only ignores reality but downgrades the patient to a commodity for convenance and profit, and clinical professionals into profit centres, required to meet ever increasing targets. This trend is diametrically opposed to the principle of ‘people‐centred care’ (World Health Organisation 2015; Hanson et al. 2022). Reductionist, metric‐driven healthcare fails many, but it is particularly discriminatory and harmful to populations whose needs are complex, stigmatised, or systematically neglected, such as older adults, people with mental health conditions, and women and girls seeking essential health services.

#### 2.3.2 Power Asymmetries and the Need for Transparency and Accountability

There is a massive power asymmetry between patient and the providers of services, due in part to the exceptional expertise acquired by healthcare professionals during their long training, in part to the fact that they are frequently arbiters of life and death, and in part because for one reason or another they have a quasi‐monopoly on the services they provide. Most of the time, this asymmetry has little consequence, and the great majority of healthcare services are delivered with professionalism and respect for the patient. However, in some healthcare systems, it is manifested in too little clinician‐patient information transfer, which can limit the ability of patients to be actively involved in decision‐making pertinent to their treatments.

Importantly, this asymmetry is also open to abuse which occasionally happens. When it does, it may be covered up, for example to protect institutional reputation (Loughnane et al. 2026). A system of selective information disclosure to patients constitutes discrimination. The patient must have the wherewithal to determine whether, if something went wrong, who was responsible and whether it could have been avoided. It is essential that every intervention is documented in a standard form, incorporated into patient records, and is accessible to the patient involved and other actors having legitimate cause, such as service hierarchies, bodies with responsibility for maintenance of professional standards and, ultimately, legal investigators, law enforcement agents, etc., who may be authorised by the patient. This would also hinder damage accumulation through repeated interventions by rare rogue healthcare professionals (Alghrani et al. 2011).

Power asymmetries are a feature of the healthcare ecosystem and in and of themselves not problematic. However, their potential for abuse must be mitigated by changes in perception of the relationships between patients and other ecosystem actors such as clinicians, from one of service provider:client to clinician:patient partnerships, and effective checks and balances, for example with much greater patient stakeholder involvement in healthcare policy and practice, as discussed below.

#### 2.3.3 Agesim and the Devaluation of Later Life

Metric‐driven healthcare services are particularly detrimental in the context of the elderly and promote a philosophy that views later life primarily through a lens of decline, dependency and cost containment, rather than holistic well‐being—encompassing physical function, mental acuity, social connectedness and purpose. This trend is diametrically opposed to the principle of ‘people‐centred care’ (World Health Organisation 2015). Truly people‐centred care must actively champion a vision of health that supports dignity, autonomy and continued contribution across the entire lifespan, fundamentally rejecting ageism in both policy and practice.

#### 2.3.4 The Stigma and Systemic Neglect of Mental Health

The foundational right to health encompasses both physical and mental health—the complete state of physical, mental and social well‐being. The accessibility and non‐discrimination clauses of the principles of universal healthcare apply equally to mental health services. This formal commitment contrasts sharply with a history of stigma and systemic neglect that have marginalised this field of medicine and its patients. Compounding this legacy, the current epidemic of neuropsychiatric conditions—particularly among young people—poses a major global health challenge. Addressing it demands that mental health receives both the priority it merits and the innovative approaches it urgently requires.

#### 2.3.5 Gender Discrimination and Violations of Bodily Autonomy

The principle of non‐discrimination applies unequivocally to all essential health services, including comprehensive sexual and reproductive healthcare—from contraception and maternal health to safe abortion care. When such care is restricted or criminalised on socio‐political rather than medical grounds, it constitutes a severe and direct violation of this right. These policies force individuals into unsafe alternatives, resulting in preventable morbidity and mortality. Beyond these measurable harms, they inflict a significant human cost, including mental trauma, unwarranted guilt, financial hardship and a fundamental disregard for personal autonomy and bodily integrity. This creates a paradox wherein prenatal health is invoked symbolically, even as systemic support for the pregnant person is withdrawn—a clear demonstration of how political imperatives catastrophically undermine the foundational human right to health (World Health Organization 2023b).

#### 2.3.6 Exclusion Through Inaccessible Design: The Needs of People With Disabilities and Variant Anatomies

A one‐size‐fits‐all system inevitably fails those whose bodies and needs fall outside a narrow, imagined ‘standard’. This includes people born with variant anatomies, those with acquired challenges from injury or illness and those managing long‐term chronic conditions. The discrimination here is often one of omission: a failure to design infrastructure, services and policies that ensure equitable participation and dignity. Healthcare systems frequently view these individuals only through a lens of acute medical ‘fixes’, neglecting their ongoing need for support, adaptations and community inclusion to lead fulfilling lives. This constitutes a systemic failure to provide the ‘complete state of physical, mental and social well‐being’ promised by the right to health.

These diverse forms of discrimination are not isolated; they intersect with those based on race, class, disability and other axes of identity, compounding disadvantages and creating significant health inequalities (Kapilashrami and Hankivsky 2018; Bohren et al. 2023). Truly people‐centred care must, therefore, be explicitly equity‐centred, requiring an intersectional lens and a fundamental rejection of these systemic biases in both policy and practice. It is also important to note that this type of entrenched inequality can be understood as a form of *structural violence*—avoidable harm caused by global and national structures that systematically deny populations the resources and conditions necessary for health, thereby prejudicing their development and dignity (Galtung 2013).

### 2.4 The Imperative to Prioritise Primary Healthcare

The World Bank (2025) has stated that ‘To reach the goal of UHC… key actions include a radical reorientation of health systems towards a primary health care approach, advancing equity in health‐care access and financial protection, and investing in robust health information systems’. The focus on hospital‐based, disease‐based and self‐contained ‘silo’ curative care models further undermines the ability of health systems to provide universal, equitable, high‐quality and financially sustainable care (World Health Organisation 2015). ‘Too many people end up in hospital, because too little is spent in the community’ (Darzi 2024). The WHO states: ‘As a foundation for and way to move towards UHC, WHO recommends reorienting health systems using a primary health care (PHC) approach’. ‘It also helps deliver the full range of quality services and products that people need for health and well‐being, thereby improving coverage and financial protection. Significant cost efficiencies can be achieved (OECD 2017) and most (90%) essential UHC interventions can be delivered through a PHC approach. Some 75% of the projected health gains from the SDGs could also be realised through PHC, including saving over 60 million lives and increasing average global life expectancy by 3.7 years by 2030’ (World Health Organisation 2025a; World Health Organization 2025b).

In spite of compelling exhortations by diverse agencies charged with promoting public health, less than half of the World's population currently has access to primary healthcare. In the face of flat or slowly‐increasing budgets, significant increases in investment in primary healthcare will require a recasting of healthcare systems driven by systems analyses of longterm needs, and creative thinking about more effective harnessing and deployment of all available resources, especially human, and agency of non‐clinical stakeholders.

### 2.5 The Issue of ‘Timeliness’: Waiting Times as an Indicator of Healthcare Accessibility and of Gaps in Health System Capacity and Performance

Extended waiting times for primary care appointments, specialist referrals, emergency responses, elective surgeries, etc., can result in preventable material deterioration in health and associated suffering or even death, plus all the associated collateral impacts on family, friends, community and their finances (Hasty 1995; Simpson 2010; Canada Commons 2015). They are also indicators of deficits in health system capacity and performance and can significantly prejudice equity (Martin et al. 2020), quality of care, and the efficiency of health systems. Waiting times in a number of countries are increasing (OECD 2020). Examples in April 2024 cited by Darzi (2024) for the National Health Service of England include:

- 1 million people waiting for mental health services.
- > 100,000 infants waiting more than 6 h to be seen in emergency departments.
- Nearly 10% of all patients visiting emergency departments had to wait more than 12 h.

Extended waiting times constitute a form of healthcare ‘rationing’ (García‐Corchero and Jiménez‐Rubio 2022) which not only result in worse outcomes in some cases, but also erodes citizen confidence and trust in healthcare systems, as patients worry whether they will receive the services they require in time and citizens wonder how much they can rely on the healthcare system in times of need. When healthcare access is restricted, it can engender views like ‘it is not worth trying to get an appointment because it will probably be impossible and thus a waste of time’ and ‘there are others worse off and more deserving than me’, even in those who are able to access health services, thereby disincentivising some of the more altruistic members of society from seeking access and thereby amplifying the problem. Waiting times are a major component of poor accessibility to quality healthcare, which is why they are at the heart of healthcare charters (https://www.alberta.ca/alberta‐health‐charter) and key determinants of public trust (Darzi 2024).

Despite efforts to reduce unacceptably long waiting times, they will occur under certain circumstances. To ensure the ethical legitimacy of such rationing decisions, frameworks like ‘Accountability for Reasonableness’ are vital. This requires that the rationale for limiting care be ‘publicly available’ and that the reasons given are ones that ‘fair‐minded’ individuals can agree are relevant and appropriate to the specific, resource‐constrained context (World Health Organisation 2015).

In some countries, a frequently employed tactic to avoid waiting times for scheduled primary care appointments is to go to the Emergency Department, which generally has an obligation to provide care. Sometimes the underlying health issues are relatively minor and this route to care adds to the already heavy burden of the Emergency Department, unnecessarily reducing its capacity to treat true emergencies (Atkinson et al. 2022; Hanson et al. 2022; Shahid et al. 2022). This must be avoided by increasing accessibility to primary healthcare.

Many health systems attempt to deal with unreasonable waiting times by setting targets. However, such targets are often based on what is perceived as readily achievable, rather than what is necessary for best outcomes, so that targets have a reasonable chance of being met and the system can declare its success. Moreover, because of insufficient systems consideration of all relevant parameters and potential collateral effects, reducing waiting times in one sphere may simply push the problem elsewhere—the phenomenon of ‘ballooning’ (Simpson 2010).

**Reducing waiting times to those which are consistent with best healthcare outcome practice must be a driving principle for healthcare resets**.

### 2.6 Healthcare Resilience: System Vulnerability, Inadequate ‘Surge Capacity’ and Decreasing Sustainability

Ensuring healthy lives and providing adequate healthcare faces many challenges, some of which are predictable, such as population growth (Ritchie and Rodés‐Guirao 2024), increased life expectancy without increased healthspan, increasing costs, poverty and malnutrition, inadequate provision of clean water, environmental pollution. Others are more difficult to anticipate and cope with in the face of limited capacity and resources, like rapidly shifting healthcare needs due to natural disasters, such as new epi−/pandemics, earthquakes and volcanic eruptions, extreme weather events, conflicts and the resulting injuries and infrastructure destruction they produce, migrations and overcrowding, often in refugee camps and informal settlements, and other causes.

Both well predicted and less well predicted challenges increase intermittent and/or long‐term resource pressures on health and social systems, be they in earlier stages of development or fully consolidated. Access to healthcare in some High‐Income Countries (HICs) may be inadequate in terms of care availability, waiting times, and affordability and catastrophic in some Low‐ and Medium‐Income Countries (LMICs). Insufficient or delayed responses to illness result in increases in disease burdens and poorer health outcomes with pertinent personal, social and economic consequences. These in turn increase the burdens on healthcare services and resources, reducing healthcare system flexibility, responsiveness and resilience.

Healthcare system vulnerability can be quantified in various ways (e.g., see Rogers et al. 2021; Behrens et al. 2022; Wickramaarachchi et al. 2025). Hospital bed occupation is one (Bosque‐Mercader and Siciliani 2023): if there is a high bed occupation, there is little ‘surge capacity’—room for unanticipated developments, crises, or armed conflicts. The COVID‐19 pandemic, which brought a number of healthcare systems to the brink of collapse, is a stark reminder of the poor resilience of many healthcare systems, an event during which limitations on resources like health professionals, beds and ventilators placed physicians in the ethically challenging position of having to decide which patients would not receive appropriate treatment and hence be abandoned to their fate.

Climate change is likely to be the next major test of resilience (see below). Another is industrial action by healthcare workers, many of whom—despite sometimes heroic devotion under difficult circumstances—are underpaid, underappreciated and lack an adequate career development pathway. This devotion is more than individual effort; it reflects a professional ‘second identity’ that health professionals assume, which entails special obligations, including a duty to put the public's interest ahead of personal interest and to report for duty during a public health emergency (American Public Health Association 2025). Sooner or later, the lack of sufficient surge capacity of many healthcare systems is likely to lead to systems failure.

The impact of health system strain is particularly severe on the most vulnerable members of society and those already poorly served by or unable to access health systems. Many, if not all, current healthcare system models may be unsustainable in the long term. While there is an increasing tendency to invest more strategically in healthcare, for example in digital medicine, there is still much investment in *more of the same*, usually to relieve individual bottlenecks—the silo approach—without recognition that more of the same is unlikely to address root causes of underlying problems. Solutions need to be sought through systems analyses, and impediments in system nodes need to be identified and addressed.

### 2.7 Climate Change Will Seriously Challenge Healthcare System Resilience and Increase Inequalities

Global warming‐caused climate change will have a massive impact on health and healthcare services in the coming years (Romanello et al. 2024; Nieuwenhuijsen 2024; Grant et al. 2025). Importantly, as people age they become less tolerant of high temperatures. Demographic changes and an ageing population result in increases in the fraction of the population most affected by a warming climate. Predictions suggest 14.5 million excess deaths due to global warming, primarily in disadvantaged regions of the world, 0.75% annual excess mortality globally, driving up health, life and casualty insurance costs, and an increase in malnourished children of 24 million by 2050. But it is increased morbidity that will account for almost 80% of the health and associated costs of global warming (with Africa and the Middle East bearing the brunt of the burden) with a cumulative projected amount of 1.27 billion Disability‐Adjusted Life Years (DALYS) and $ 2.7 trillion in lost productivity and healthcare costs (World Economic Forum 2024).

In addition to the inherent heat susceptibility of older members of society and those in poor health, workers employed outside in agriculture and construction are particularly exposed to extreme weather, primarily high temperatures. A defining characteristic of the human species is the extent to which it orchestrates its own living conditions, for example through dwelling construction, urban settings, etc., and its interactions with the environmental conditions of the natural world, through clothes, etc. Most humans spend most of their time in environment‐controlled settings: homes, schools, workplaces, centres of worship, culture and leisure, modes of transportation, etc. However, most of these have not been designed or constructed to function as planned in weather extremes of the type being increasingly experienced. To achieve the same level of control characteristic of times when weather events were less extreme, new construction and retrofitting actions are necessary. This will require a significant increase in construction activity, purely to adapt to climate change. However, precisely this need exposes the people who can satisfy it to health debilitation by such weather extremes. Climate change will thus simultaneously raise the need for increased construction activity and cause reductions in construction worker health and availability. Since the construction industry is a major producer of greenhouse gases (United Nations Environment Programme 2024; https://www.architecture2030.org/why‐the‐built‐environment/) and contributor to global warming, this constitutes a vicious cycle.

A similar scenario will play out in the sphere of healthcare: the health‐debilitation effects of climate change will both increase the need for healthcare services and directly affect the health of healthcare professionals, especially the older and more vulnerable members, and those working in environments that are less well adapted to weather extremes, including some hospitals and health centres. Home‐visiting care workers who spend considerable amounts of time travelling will also be increasingly affected by weather extremes. Thus: climate change will raise healthcare needs and simultaneously reduce the availability of healthcare staff, as was experienced in the COVID‐19 pandemic (British Medical Association 2022).

One estimate suggests that climate change‐caused reductions in worker availability in the food and agriculture, construction and healthcare industries will cost a total of $1.5 trillion by 2050, much of it ($1.1 trillion) within healthcare (World Economic Forum 2025). Without a radical reset of healthcare systems, climate change is very likely to engender a worsening relationship between healthcare capacity and need that will dramatically increase strains on healthcare provision and accessibility, perhaps in some cases causing system collapse. These strains will preferentially impact the more disadvantaged members of society and hence increase inequalities.

## 3 Solution Strategies Grounded in the Principle of Best Health Outcome Practice

### 3.1 Healthcare Resets: The Need for System Analysis and Integration of All Parameters Impacting Health in Consideration of Solution Strategies

Healthcare systems have largely evolved organically, responding to new problems (such as changes installed following the COVID‐19 pandemic) and new discoveries/opportunities (such as the explosion in non‐invasive imaging diagnostics), in part independently of non‐medical parameters that affect health. Moreover, commercial providers of diagnostic tools, prevention measures, and treatment options have mostly pursued commercial goals, rather than the priority needs of healthcare systems—witness the almost empty antimicrobial development pipelines in favour of other pharmaceuticals perceived to have greater revenue potential (Anderson et al. 2023). As a result, there are significant imbalances in healthcare capacities and there is an urgent need to align research and development and commercial innovation with medical needs (National Academies of Sciences, Engineering, and Medicine 2025) and thereby increase healthcare system resilience. Successful historical precedents like antibiotic discovery, vaccine development (including the recent COVID‐19 vaccine) and cancer research clearly demonstrate the political ability to effectively align healthcare service innovation and needs through priority setting and funding.

A systems analysis must also incorporate the fact that healthcare ecosystems are dominated by self‐reinforcing vicious cycles. These include: *increasing longevity without proportional gains in healthspan*, which raises complex demand; *global warming*, which simultaneously increases healthcare needs and debilitates workforce capacity; *rising costs driven by new, often hospital‐centric technologies*, which divert resources from primary and preventive care; and the *privatisation of services without strict equity controls*, which prioritises profit over access, eroding public resilience. Each cycle amplifies system strain, creating feedback loops that accelerate towards crisis. A reconstituted system must therefore be designed to trigger *virtuous cycles*: where patient empowerment and digital tools increase healthcare access, unlock professional capacity and improve disease outcomes, where frugal microbial technologies lower costs and increase deployment and access, and where investment in prevention and primary care reduces downstream hospital demand, freeing resources for further innovation and equity. **The fundamental task of reform is to identify and decisively interrupt vicious cycles while strategically instigating virtuous ones**.

Re‐sets must be based upon a systems analysis of what is essential for future needs, challenges and sustainability (see, e.g., Smith et al. 2023), not that which is readily available or easily attained, with the goal of deploying resources for maximal efficiency and patient benefit. System perspectives, such as those inherent in the Manhattan Principles and One Health (Wildlife Conservation Society 2024) and Health in All Policies (World Health Organisation 2014; Greer et al. 2023) paradigms, the role of the microbiosphere (Timmis, Baquero, et al. 2025) in both of these, and the development and exploitation of effective digital frameworks, are urgently needed. Importantly, healthcare can no longer be restricted to physicians, medical centres and the pharmaceutical industry: a holistic approach that comprehensively integrates these with disease prevention, hygiene, lifestyle, work, nutrition, water treatment, environment, pollution, inclusive design, self‐help and education, is needed (Timmis and Hallsworth 2024).

Crucially healthcare systems must be governed by the principle of ‘Networked Agency with a Safety Net’. This means the digital transformation and patient empowerment essential for future resilience must be consciously designed to avoid a libertarian erosion of collective health responsibility. Artificial intelligence (AI) and integrated data systems must act as intelligent mediators, enabling distributed action and patient agency while ensuring continuous, systemic oversight and safeguarding public health imperatives (see also Armoundas and Loscalzo 2025). It must also be guided by a positive philosophy of health and ageing that values physical and mental well‐being, autonomy and social participation across the entire life course, actively combatting ageism and stigma in both policy and practice. Healthcare systems must be *reconstituted* to:

- Have sufficient capacity to promptly satisfy healthcare service needs.
- Be stress resilient and able to manage unanticipated increases in demand without significant impact on service provision.
- Be patient‐centric (World Health Organisation 2015; Hafiz et al. 2024), not profit‐centric.
- Be led by physicians aided by administrators, not the other way round.
- Be societally equitable.
- Improve logistics to enable more frequent patient, healthcare professional and diagnostic‐treatment‐monitoring instrumentation contacts in optimal time and space, with universal design principles ensuring full access for people with disabilities and other anatomical challenges.
- Be environmentally sustainable in design and operation, ensuring that new and repurposed healthcare infrastructure is sited at public transport nexuses, constructed or retrofitted using low‐carbon and circular materials, and powered by renewable energy systems where feasible, thereby reducing the system's direct and indirect carbon footprint.
- Be underpinned by the 7 I's: *Investment in Information technology, Innovation (especially in frugal technologies), Immunisation (including development of effective vaccines for neglected tropical diseases), Incentivisation (of healthy lifestyle, education), Insurance (equitable, universal) and Inclusion*.

The last ‘I’, Inclusion, demands the involvement of patients and patients‐to‐be, as major stakeholders in healthcare, in discussions about relevant elements of policy, including priorities, organisation and administration of healthcare, and especially in the recasting of healthcare systems that we discuss in this paper. Inclusion is a key element of good governance (see also Petkovic et al. 2023; World Health Organisation 2014). As stated by the United Nations Educational Scientific and Cultural Organization (2009) ‘…governance is …‥ about power relationships in society. At its most basic level, governance systems define who decides on policies, how resources are distributed across society and how governments are held accountable’. The inadequate and infrequent inclusion of patients as core stakeholders in healthcare strategy‐policy development urgently needs correction.

### 3.2 Healthcare Resets: Integrating and Decisively Raising Key Barriers to Disease

Over recent years, there has been a considerable shift in healthcare emphasis from disease treatment to prevention (Caron et al. 2023; https://www.emro.who.int/about‐who/public‐health‐functions/health‐promotion‐disease‐prevention.html). Nevertheless, the major focus in most systems is still the sick patient with corresponding disproportionate investments in hospital ecosystems (Darzi 2024). A considerably increased emphasis on disease prevention would have major beneficial impacts on a range of societally‐vital parameters, including healthcare costs, physical and psychological suffering, lost time at school and the workplace with resulting reductions in benefits from education, training, continual professional development and re‐skilling, collateral consequences such as loss of productivity, the need for carers, and mental, physical and economic strains on family‐social support networks.

Disease prevention is traditionally viewed through the clinical lens of vaccines, pre‐emptive surgery and other treatments. However, since disease and suffering have many different causes, prevention in future must be viewed through the much wider lens of all parameters that can influence health—physical and mental (Timmis, Karahan, et al. 2025)—basic services, nutrition, behaviour‐lifestyle, environment including the built environment, employment, addictions, social environment including pressures of social media on the young, and mobility‐friendly environments for the disabled. Key barriers to disease that must be raised decisively include:

#### 3.2.1 Basic Goods and Services

Many individuals across the globe, especially those residing in rural communities in low‐income settings, have suboptimal access to facilities for sanitation, clean drinking water, and healthy nutrition; some communities live in heavily polluted environments. Such conditions predispose people to disease in general, and infections in particular. Microbial technologies, such as those underpinning wastewater treatment, drinking water purification, pollutant treatment at source, bioremediation of polluted environments, food production and safety, can hugely contribute to improvement of all these deficits (see Anand et al. 2023, for a more comprehensive overview), so the decision is one of commitment to strategic investment in LMICs, especially by HICs, International Organizations, Non‐Governmental Organizations and philanthropic agencies. The issue of health‐relevant basic services is discussed in more detail in an accompanying Editorial in this Special Issue (Timmis, Karahan, et al. 2025).

#### 3.2.2 Diagnostics

Early diagnosis of disease can lead to interventions that result in improved outcomes (Hasty 1995) and that, in turn, reduce burdens on healthcare resources. The same is true of early diagnosis of some predisposing factors like genetic, physiological or existing health issues. To maximise such improved outcomes/burden reductions, it is essential to promote expansion of the range of diagnostics available, particularly with an eye to simple‐to‐use diagnostics for point‐of‐care (POCT) applications and self‐diagnosis—always with clinical oversight (Timmis and Timmis 2017). For equitable healthcare access, it will be essential to emphasise the development of frugal diagnostics that are affordable in low resource settings (Anand et al. 2023; Timmis, Karahan, et al. 2025), and to develop new diagnostics to inform on health conditions that are relevant to low resource settings (e.g., for neglected diseases). The basis or production of many diagnostics involves microbial technologies (Timmis and Hallsworth 2024).

#### 3.2.3 Vaccines

One of the most effective measures to prevent infections is the use of vaccines, which have prevented billions of infections and premature deaths (https://www.who.int/news‐room/spotlight/history‐of‐vaccination/a‐brief‐history‐of‐vaccination; https://immunizationdata.who.int; https://worksinprogress.co/issue/the‐golden‐age‐of‐vaccine‐development/). Vaccine production is a classic microbial technology that continuously undergoes creative innovations, as witnessed recently during the COVID‐19 pandemic (National Institutes of Health 2020). The property of herd immunity—the limitation of disease transmission (the R_0_ number) achieved by reducing the proportion of susceptible individuals in a population through comprehensive vaccination campaigns—is central to some vaccination strategies and, in some cases, can lead to disease elimination or eradication, regionally or globally (Fine et al. 2011). However, elimination can be reversed under certain circumstances. During the last decade, a number of countries were declared free of measles, only to lose this status more recently as a consequence of falling vaccine rates (Najera 2025).

Vaccines for infections most relevant to HICs are widely available (Sabo et al. 2024), but for some infections pertinent to LMICs, especially neglected tropical diseases (World Health Organisation 2024), vaccines are only slowly being developed (Anderson 2021; Chavda et al. 2025). Such vaccine needs must be urgently prioritised.

While vaccines currently represent our most powerful disease prevention measure, vaccine hesitancy among individuals and societal groupings is a phenomenon challenging both the protection of individuals and the protection of community members through herd immunity, especially vulnerable members with poor immune responses because of underlying health issues or ongoing therapies who cannot be protected by vaccination. The WHO declared that vaccine hesitancy is one of the 10 threats to global health (World Health Organization 2019).

Recent research reveals an association of socio‐political views with vaccine hesitancy and indicates the need for a different approach to vaccination campaigns that consider the granularity of attitudes of those addressed by the campaigns and the spread of misinformation (Jäckle and Timmis 2024, 2025). Addressing this challenge requires strategies that respect individual autonomy while upholding collective responsibility—a balance that can be dynamically managed through the AI‐mediated, data‐informed public health capabilities of the proposed National Clinical Informatics Centre (NCIC; see below), enabling targeted, effective interventions.

#### 3.2.4 Healthier Lifestyles: The Role of Education

An effective but thus far underexploited means of relieving pressures on healthcare systems is disease reduction through health creation policies, measures and practices (Katz and Ali 2009). Since lifestyle habits are often influenced by environment, efforts to elevate health in the population must include education at all levels, but particularly at school.

While almost everyone knows that smoking, drug and alcohol abuse, sedentary lifestyles, over‐eating and consumption of ultra‐processed food, among others, predispose to disease, many do not change their habits, at least until it is too late. Many children grow up in poverty and experience poor nutrition and limited education, which often results in poor development and health. Other children grow up in affluence which may be associated with unhealthy eating and bad behavioural practices, lifestyles that are often propagated in adulthood. It is essential to counter though education in childhood the lack of understanding of the importance of lifestyle for good health in childhood and adulthood, for the realisation of ambitions, and for satisfaction in social interactions (e.g., obesity, like other health issues, is often stigmatised, prejudicing social interactions). This education must also address lifestyle's profound impact on mental well‐being. It should dismantle stigma, promote emotional literacy, and highlight the vital connections between mental and physical health, such as the gut‐brain axis.

Education of children about mental health and disease is key to their adopting practices and attitudes that are beneficial for themselves and people in their networks. Such education must also reveal the wider context: the consequences of individual behaviour and attitudes for the wider community, and the importance of the environment—One Health—as a health parameter. Health education is of vital importance for effective personal engagement in one's own health (see below).

Above and beyond this, education about health and disease is essential for several reasons, including understanding how infectious diseases are transmitted and what actions can reduce transmission, including those that may impact personal liberty in the case of epi/pandemics, and especially the issue of sexually transmitted and drug abuse‐associated diseases. When public health actions restrict liberty, the ethical principle of reciprocity obligates authorities to ‘offset the potential harms and losses’ imposed on individuals (American Public Health Association 2025). For example, if a system mandates quarantine, it bears the responsibility to provide medical assistance, housing and nutrition, ensuring a mutual exchange of obligation.

#### 3.2.5 Access to Comprehensive Sexual and Reproductive Health Services

As a fundamental component of non‐discriminatory healthcare (see above), universal access to comprehensive sexual and reproductive services is a cornerstone of preventive health and a formidable barrier to disease. Where these evidence‐based services—including contraception, maternal healthcare and safe abortion—are restricted, inaccessible, or criminalised, they create a direct and severe barrier to health. This leads to preventable mortality and morbidity, entrenches gender‐based socioeconomic inequalities and constitutes a direct manifestation of structural violence (Galtung 2013). Research and policy in this area must therefore actively unmask and challenge the power structures that restrict rights, moving beyond technical solutions to address the political and social determinants of health (Schaaf et al. 2021; Kapilashrami 2020). The provision of these services is non‐negotiable for equitable, patient‐centric care.

#### 3.2.6 Aligning Medical Knowledge Creation and Clinical Advances With Global Health Needs

A fundamental barrier to equitable healthcare is the structural disconnect between the creation of medical knowledge and its translation into clinical practice, on one hand, and the global distribution of health needs, on the other. Biomedical research priorities, largely set in High‐Income Countries (HICs), are influenced by commercial potential, academic interest and regional disease burdens. This results in an innovation pipeline that is often misaligned with the conditions causing the greatest morbidity and mortality in Low‐ and Middle‐Income Countries (LMICs), such as neglected tropical diseases and context‐specific health challenges. Furthermore, the definition of an ‘equitable level of healthcare’ is not merely a technical question of resource allocation, but a socio‐political one that engages with differing cultural expectations of health, well‐being and the role of medicine. Universal health coverage cannot be achieved simply by distributing HIC‐centric technologies. It requires a fundamental decolonisation of the global health research agenda, reforming funding processes to support priority‐driven research in LMICs and recognising the epistemic injustice inherent in current systems (Garrafa et al. 2010; Ashuntantang et al. 2021; McCoy et al. 2024; Koum Besson 2022). The persistent misalignment between research priorities and global disease burden constitutes a form of structural violence (Galtung 2013), where institutional and economic structures actively prevent the creation of knowledge and tools needed to address the basic health needs of the most vulnerable populations. This realignment is a prerequisite for true equity.

### 3.3 Unlocking Resources and Capacity: Increasing Deployment Effectiveness of Healthcare Actors

#### 3.3.1 Healthcare Professionals

Clinician availability is often a bottleneck in healthcare accessibility (e.g., Melford‐Davis and Malani 2024; Walensky and McCann 2025). To become a qualified physician requires many years of university and hospital study and training that make physicians some of the most highly trained professionals on the planet. The financial, time, and resource costs are enormous. However, once qualified, many spend part of their time diagnosing and treating routine conditions and associated bureaucratic exercises: this represents a significant underuse of talent and investment. According to the Independent investigation of the NHS (National Health Service) in England, from 2019 to 2023, surgical activity per surgeon decreased by 12% and activity of clinicians working in emergency medicine fell by 18%. This fall in productivity of frontline staff caused by diversion from primary duties causes significant disengagement (Darzi 2024). Such personal frustration of not being able to exploit full potential can lead to loss of clinical professionals from the system, either through a move to other health systems or even to other professions, thereby exacerbating the problem of clinician availability.

In many countries, there are either significant backlogs in treatment of more complex, severe and urgent medical conditions, backlogs that could be reduced if physicians were freed from routine tasks and bureaucracy, or commercial obstacles preventing treatment of patients who cannot pay. This constitutes an unnecessary but substantive burden on healthcare resources, in this case physicians. There is sometimes a disconnect between on one hand what patients need and what physicians can do to satisfy such needs, and on the other what physicians actually do. There is an urgent need to release highly qualified medical practitioners from responsibilities that could easily be fulfilled by others to enable them to realise their full potential and play optimal roles in healthcare and reduce the burden on those currently dealing with complex and urgent medical conditions. Ensuring this paradigm shift is ethically grounded requires that ethics education be a ‘central part’ of ongoing training to help practitioners identify ethical dimensions in their work, and that ‘formal structures, such as ethics committees’ be established to provide a deliberative forum for complex decisions (American Public Health Association 2025).

There is also often a shortage of other healthcare professionals, like nurses and technicians (Kumar et al. 2025). Bureaucratic tasks can also reduce the availability of these professionals, and similar options to counter this are available.

This dynamic is further complicated by the persistent ‘brain drain’, where health professionals trained in resource‐poor countries are recruited to wealthier nations, a process exacerbated by funding models that prioritise HIC institutions and create significant indirect costs for underfunded foreign partners (Crane et al. 2018). This represents a significant ethical conflict between the individual ‘freedom to relocate’ and the global obligation to improve the health of the most vulnerable people, and perpetuates a *status quo* that entrenches inequality (World Health Organisation 2015).

#### 3.3.2 Patients

To a very considerable degree, patients are passive bystanders in their health issues. But patients have a vested interest in best possible outcomes, and many have the potential to contribute actively in the prevention, diagnosis and treatment of their disorders. Moreover, targeted education could hugely increase patient abilities to participate actively in their own health. The need for public education‐training in some basic healthcare procedures is recognised by compulsory first aid courses for driving licences in some countries and the general prevalence of facultative courses for the general population. Indeed, a central plank of the well‐established Chronic Care Model for management of a range of different chronic diseases is patient self‐management, supported by education both in specific knowledge and technical skills, and—importantly—in problem solving (Bodenheimer 2003).

It is crucial to change perceptions of patients and view them as potential actors in healthcare, as partners rather than clients of health professionals, and to vigorously pursue strategies, incentives and enabling measures to actively engage them in their own health in situations in which they can contribute usefully (Armoundas and Loscalzo 2025; Power 2025). Promoting patient agency will enable reattribution of health professional time and a better matching of skills to healthcare needs. This in turn will slow cost increases and raise patient and clinician satisfaction.

A simple example of this is the repeated recording of patient details over lifetimes. This is usually done by healthcare professionals, often by nurses or sometimes a clinician, sometimes repeated multiple times by different actors along a diagnosis‐treatment pathway, thus amplifying the time taken by professionals to query information that many patients themselves are able to provide on‐screen. AI‐assisted querying of relevant information for different health issues, inputting by patients, and centralisation of patient data linked to unique identifiers, such as that provided to researchers by ORCID (https://orcid.org), would reduce costs and save much time that could be redeployed for essential duties. Moreover, because all relevant information is not necessarily recalled for each new visit to the doctor, especially if the reason is associated with significant discomfort and stress, and because memory tends to fade with age, patient inputting of information would involve only updates after the initial account creation, thereby also preserving early health‐relevant information and eliminating the effort (and frustration) of repeatedly providing the same information.

Moreover, there are a number of routine tasks in primary healthcare, especially in terms of diagnosis, as is the case with pregnancy tests and SARS‐CoV‐2 lateral flow tests, and of treatment, as is the case with routine injections of insulin and weight‐loss drugs, where patients themselves can satisfactorily perform key tasks, thereby further relieving the burden on health professionals. This could be significantly increased, resulting in substantive reductions in the commitment of trained personnel, if determined effort were made to advance enablement of patient involvement in their own healthcare. The motivation of patients to actively participate in their own care can in most cases be expected to be high; familiarity and comfort with digital interfaces will also be high among younger patients.

Advancing patient agency will involve:

- Increased deployment of existing, and accelerated development of a much greater range of new simple POCT tests, such as those based on lateral flow, LAMP, etc., systems, and the miniaturisation of existing ones. Many of these are either microbially based (e.g., LAMP uses the *Bst* polymerase), or components are produced in microbial cell factories;
- Expanding the availability and functionalities, and reducing the cost of smart wearables;
- Establishing effective and user‐friendly digital interfaces; and
- Creating simple, low‐cost professionally supervised healthcare venues that provide more advanced diagnostics usable by patients (see below).

There is a need for public:private partnerships with identification of frugal simple‐to‐use products of strategic importance, the development of which must be incentivised. In all such partnerships, there is an ethical responsibility to ensure that work is not ‘unduly influenced by secondary interests’. This requires establishing transparent mechanisms, such as ‘periodic financial disclosures’, to manage organisational conflicts of interest and maintain public trust (American Public Health Association 2025). Healthcare policy and governance must steer evolution towards current and future patient needs and sustainability with much greater determination and clarity of purpose.

### 3.4 Unlocking Resources and Capacity: More Cost‐Effective Use of Healthcare Facilities: The Need for Lower Cost Facilities for Placement and/or Re‐Location of Patients Not Requiring a High‐Cost Clinical Environment

Although there is a considerable diversity of healthcare facilities that also vary considerably between countries and systems, a common denominator is the doctor's surgery/community clinic, which provides primary healthcare by general practitioners, and the hospital, which provides secondary and tertiary healthcare by specialist clinicians. Specialists may also have their own private clinics, as do those engaged in dentistry, physiotherapy, psychotherapy, etc. Primary healthcare centres tend to have modest diagnostic‐treatment instrumentation and to be relatively ‘low cost’ facilities. Hospitals, on the other hand, tend to have highly sophisticated diagnostic‐treatment instrumentation requiring highly trained personnel and, in some cases, special housing infrastructure. Beds are usually in rooms disposing of sophisticated monitoring infrastructure. Operating theatres and intensive care suites have very high levels of monitoring facilities and hygiene control. Hospitals are ‘high cost’ facilities. However, this binary system is rarely cost effective because hospital stays often include healthcare that does not warrant or require high levels of medical technology.

Creating more high technology/high‐cost facilities, while beneficial and promoting ever more advanced interventions, and hence newsworthy creating opportunities for news coverage and photoshoots for health ministers, diverts funding away from the more urgent basic needs of many that can be addressed by more frugal options. In future, it is essential that reducing costs and benefiting the many also become newsworthy.

Availability of alternative lower‐cost facilities would decrease pressure on bed occupancy in ‘high cost’ facilities and increase availability of high technology/care for patients needing them. It would also significantly increase healthcare system resilience and should, in the long term, reduce per patient healthcare costs.

We envision three types of additional healthcare facilities:

- low‐cost hospitals with basic technology for patients with conditions that can be monitored, diagnosed, or treated with instrumentation that requires little in the way of infrastructure, or for patients with low risk of complications, such as some that are recuperating from treated diseases, or for quarantining or isolating people having had exposure to a transmissible infection, as was the case during the COVID‐19 pandemic (Timmis et al. 2020),
- ‘Do‐It‐Yourself’ primary healthcare clinics: Digital Medical Centres, and
- ‘Home Clinics’.

Importantly, all such new facilities must be conceived and implemented under a core design principle of environmental sustainability. This mandates their strategic siting at public transport nexuses to minimise patient travel emissions, the use of adaptive reuse (e.g., of underused retail or commercial spaces) or construction with low‐carbon, circular materials and the integration of energy‐efficient systems. This approach ensures the healthcare system's physical footprint actively contributes to planetary health and community resilience.

### 3.5 Unlocking Resources and Capacity: Increasing Access to Primary Healthcare Through Digital Medical Centres and Home Clinics Conceived and Designed for Patient Agency

We have previously described potential scenarios for two types of clinically supervised venue for active patient involvement in their own healthcare: *The Do‐It‐Yourself (DIY) Digital Medical Centre* (Timmis and Timmis 2017) and *The Home Clinic* (Timmis et al. 2019). These models of self‐care operate under the governing principle of ‘Networked Agency with a Safety Net’, where patient autonomy and action are enabled and safeguarded by clinical oversight.

#### 3.5.1 Do‐It‐Yourself (DIY) Digital Medical Centres

DIY Digital Medical Centres are local, readily accessible walk‐in venues open 24 h per day, 7 days per week. They are virtually autonomous, clinically supervised primary healthcare centres, linked in real time to a *Patient Data Centre* and an *e‐clinician centre*. They are designed primarily to enable patients themselves to carry out simple diagnostic‐monitoring‐treatment tasks in response to AI and remote clinician instructions and thereby reduce pressure on primary healthcare services. They are supported by onsite clinical professionals and a pharmacy around the clock (for a detailed description of the strategic aims, *modus operandi* and expected benefits of the DIY Centres, see Timmis and Timmis 2017). Patients would:

- Create standardised patient documentation in response to precise querying by the *Patient Data Centre* digital interface.
- Perform clinically‐supervised routine diagnosis‐monitoring‐follow‐up procedures (see also Kumar et al. 2025), with the aid of digital and, where necessary, professional guidance, the results of which would transmit in real time to the *Patient Data Centre* and *e‐clinician centre* for clinical assessment.
- Access confidential, initial mental health triage and support sessions via private telemedicine booths linked to the e‐clinician centre.
- Immediately receive treatment recommendations and prescriptions or, where necessary, instructions to go to a conventional healthcare venue for a more specific diagnosis or treatment.
- Receive recommendations concerning check‐ups and appointments with health professionals.
- Receive personalised health and lifestyle information and advice.

DIY Centres would not only be used for self‐diagnosis by patients, but also for routine interventions like vaccinations by on‐site health professionals, and other routine public health activities as needed. They would constitute community ecosystems for health information and engagement and serve as venues for health‐ and healthcare‐relevant education.

An essential characteristic of DIY centres is accessibility by the communities they serve by affordable, frequent and well‐networked public transport. They are local. However, there are multiple options for their integration into town healthcare ecosystems ranging from small, stand‐alone centres to those acting as healthcare hubs in large multifunctional ‘one‐stop’ primary healthcare centres (see below).

DIY Centres would be linked to

- a national *e‐clinician Centre*, a telemedicine centre staffed 24 h per day, 7 days per week by clinicians accessing patient test results from the DIYs in real time, patient data from the *Patient Data Centre*, and communicating directly with patients and on‐site health professionals. e‐Clinicians would not only be experts in AI‐empowered telemedicine but, to effectively communicate with all patients, also collectively cover the principal languages spoken in their country (see below: *the need to join up*)
- a *National Clinical Informatics Centre* (NCIC), responsible *inter alia* for developing AI‐algorithms for patient interfacing in DIY Centres and clinical diagnosis, collecting and analysing clinical and clinically‐relevant data, developing personalised treatment strategies (precision medicine) and predicting treatment outcomes, formulating and updating best health outcome practices, documenting health trends in the population, and much more (see Timmis and Timmis 2017, for a more detailed description of the multitude of roles of the NCIC). It would also provide via a dedicated website best available health information to the healthcare professions and advice to the public, thereby countering some poor and inaccurate—often commercially‐motivated—information currently provided by the worldwide web. The NCIC consists of a cluster of highly integrated and synergistic centres, namely
  ○ the *Patient Data Centre*
  ○ the *Population Trends Analysis Centre*, which analyses patient information fed into the Patient Data Centre and focuses on epidemiology and trend recognition and analysis. It is responsible for identifying new disease trends and monitoring population‐level mental well‐being metrics and emerging mental health crises, and establishing early‐warning systems for disease emergencies. Practitioners operating such a centre must navigate the different ethical requirements for public health surveillance (active monitoring) and research (knowledge creation), especially since surveillance is often ‘nonconsensual’ and requires special attention to individual privacy and confidentiality (World Health Organisation 2015). This is particularly critical for AI applications in sensitive areas like sexual and reproductive health rights (SRHR; United Nations Human Rights 2018; Organisation for Economic Co‐operation and Development 2025) where research must move ‘beyond no harm’ to establish robust ethical frameworks for data governance and prevent algorithmic bias and stigmatisation (Tamrat et al. 2025).
  ○ the *Biomedical Knowledge Hub* (BKH), which detects and assesses the relevance for clinical practice of latest biomedical discoveries and, where appropriate, integrates these into practice/recommendations. It also continuously assesses clinical needs and integrates these into research priority policy recommendations, including the need for specific clinical trials and how they should be conducted. The BKH would therefore play a pivotal role in maintaining medical practices at the forefront and influencing research policy to reflect clinical needs. In so doing, it would provide a counterbalance to current research priorities which sometimes reflect personal interests and the issue of *what can easily be done* as opposed to *what needs to be done*.
  ○ the *Policy Innovation and Evaluation Centre* integrates policy‐relevant activities of the other centres to develop recommendations for new policies, regulations, practices and benchmarks. This includes recommendations for incentivisation of development of new diagnostics and diagnostic equipment, therapies, etc. that may not be immediately commercially attractive

In essence, the NCIC institutionalises a continuous, evidence‐based conversation between individual health actions and collective health outcomes, using AI to translate data into dialogue, ensuring personal agency and public responsibility are constantly aligned. It actively steers the evolution of the healthcare system.

#### 3.5.2 The Home Clinic

The Home Clinic is a simpler, personal 24 h per day, 7 days per week version of the DIY Centre that uses the home computer, tablet or smart phone as the digital interface with the *Patient Data Centre*, the NCIC and the *e‐clinician centre*, and reduces pressure on primary healthcare services. It relies upon a certified delivery service to provide (and, where appropriate, recover) the diagnostic tests, equipment of varying complexity and, where necessary, medicines from local or regional suppliers (Timmis 2020). Like the DIY Centre, it can provide all manner of clinically supervised services (prescriptions, appointments, reminders, personalised health and lifestyle advice, etc.), except those requiring an onsite health professional. The use of clinically approved Apps will also play a key role in the Home Clinic healthcare ecosystem. *The Home Clinic constitutes a reversal of the normal practice of the patient going to the healthcare centre because, in this case, healthcare comes to the patient*.

In terms of improving access to healthcare, the subject of this element of SDG 3, it is important to note that the creation of DIY Centres and the Home Clinic

- provides essentially unlimited access 24 h per day, 7 days per week to primary healthcare because they are digitally centric,
- eliminates regional disparities in healthcare access—the time–space–clinician availability‐funding issue (the ‘*postcode lottery*’)—by concentrating physicians expert in digital medicine in a few linked centres that provide universal coverage around the clock,
- allows highly trained health professionals to deal with more cases really requiring the skills they have learned over their professional lives, by relieving them of the burden of simple routine activities (see also Kumar et al. 2025),
- reduces costs and frees up key infrastructure for those more in need by transferring patients from overburdened high cost‐high technology clinical settings to low‐cost settings

Healthcare service provision in the home is of course nothing new: home visits by general practitioners were common in the past, and ‘hospital at home’ and ‘virtual wards’ (Norman et al. 2023; Hospital at Home 2023) have been explored in various versions, the latter driven to some extent by the COVID‐19 pandemic (Richards et al. 1998; Leong et al. 2021). Virtual wards, while reducing pressure on hospitals and their infrastructure and undoubtedly beneficial to some patients, have so far had mixed success (Oliver 2023; Co 2024). In any case, they are very different from the Home Clinic concept proposed here which is self‐help, primary care, and telemedicine‐centric.

### 3.6 Unlocking Resources and Capacity: The World of Health Apps and Wearables

The Home Clinic exists side‐by‐side with the digital world of health Apps, smart phone/wearables Apps that can monitor an increasing range of health parameters and provide information and advice on such issues as fitness tracking, nutrition and diet, mental health and mindfulness, chronic disease and rehabilitation management, sleep patterns, women's health and telemedicine. The data collected, its analysis, and advice transmitted by different Apps can be of variable quality. Digital mental healthcare may currently represent the field experiencing the greatest use of Apps. While currently prominent in mental health, the utility of clinically approved Apps for therapy, mindfulness, and mood tracking within the safeguarded Home Clinic ecosystem could be transformative, provided they feed into the NCIC's oversight for quality and safety.

While Apps undoubtedly have enormous potential for self‐care in the context of the Home Clinic, and of reduction of patient burden on healthcare systems, professional medical oversight and quality control of data generation, analysis and feedback are essential. A more global utility of Apps‐wearables and their predictive power will depend upon their feeding standardised quality‐controlled information into national data centres, like the NCIC outlined above (see also Barker et al. 2024; Armoundas and Loscalzo 2025).

Although professional medical oversight and quality control of data generation, analysis and feedback are essential, the net environmental impact of this digital ecosystem is beneficial. While digital infrastructure has an energy cost, its ability to prevent countless physical trips and enable precise, targeted care results in a substantial net reduction in the carbon footprint of healthcare delivery.

### 3.7 Clinical Oversight of DIY Healthcare and the Issue of Overdiagnosis

We have emphasised the importance of increasing early diagnosis and treatment of disease to decrease disease incidence and severity in the population, and its associated financial and human suffering costs, through patient participation and the use of easy‐to‐use POCTs, etc. However, precisely for the same reasons of cost and human suffering reduction, it is important to avoid overuse of diagnostics/overdiagnosis which may not be clinically indicated, and hence constitute an unnecessary financial cost (OECD 2017), and may prompt unwarranted anxiety in the patient. For this reason, all DIY activity must be carried out in the context of appropriate clinical oversight and conform to good clinical practice. This oversight should be supported by formal ethics committees, which provide a vital deliberative forum to address ethical disagreements and ensure decisions are not arbitrary, especially in the context of patient‐led care (American Public Health Association 2025).

### 3.8 Unlocking Resources and Capacity: Incentivisation of Development Simpler, Mobile Instrumentation to Increase Patient Access to Healthcare

Exciting research discoveries and their development lead to increasingly sophisticated diagnosis‐treatment technologies characterised by greater precision, sensitivity and adaptability to patient‐disease diversity, or an improved ability to manage rare health conditions, which is vitally important for medical progress. However, in the short term they often benefit only a small group of privileged individuals. Moreover, their newsworthiness and the excitement this generates may divert attention from other types of innovation, particularly the development of simpler, less sophisticated and less costly technologies that can immediately benefit large numbers of patients and, in some cases, provide services previously only available to a few, thereby reducing or eliminating a source of inequality.

An increasing number of simple POCT tests enable diagnoses to be carried out almost anywhere, which is important for rural health centres in low resource settings. Many diagnostic and monitoring procedures currently involve imaging. Ultrasound examination is one of the most common procedures, and ultrasound instruments are relatively small and mobile (indeed, there are basic probes that can be linked to a smart phone which can display the images). There are also mobile, low power‐consuming MRI systems that can be used for head imaging. These can in principle not only be used at scale in locations far from major healthcare centres but also be readily transported between such locations to increase availability‐accessibility, satisfy needs and assure maximal exploitation.

Low‐cost facilities will be able to provide an increasing number of healthcare services for those patients not needing a high level of infrastructure. What is needed is increased selective investment in and incentivisation of the development of new POCTs, mobile imaging systems and other instrumentation that can function in low‐cost settings.

### 3.9 Unlocking Resources and Capacity: Joining Up Fragmented Healthcare Services

There are several forms of fragmentation in healthcare ecosystems, all of them important contributors to inadequate accessibility to quality healthcare, which include siloed patient data, fragmented healthcare services, fragmentation of travel options and location of healthcare services, and healthcare professional:patient communication and understanding. Fragmentation is particularly challenging for patients with multimorbidities (Prior et al. 2023).

#### 3.9.1 Patient Data—Joining Up Patient Data and Clinicians

Some healthcare ecosystems use multiple patient data acquisition and handling systems, some of which silo the data, complicating interoperability and optimal utilisation by different clinical actors (e.g., Chen et al. 2024; see also Pistoia Alliance‐ZS 2024). We have proposed that a *National Patient Data Centre* be created for the acquisition and handling of all patient data and for making it available to authorised clinicians. The Patient Data Centre would be embedded in the *National Clinical Informatics Centre*, which would be responsible for developing systems and AI‐algorithms and setting and imposing standards nationally to eliminate data siloing and to ensure interoperability. Critically, this secure, unified data infrastructure is the essential foundation for the ‘Networked Agency with a Safety Net’ model. It is what makes safe decentralisation possible, allowing for patient empowerment through DIY and Home Clinics while ensuring continuous, intelligent oversight and a robust safety net provided by the healthcare system (see Timmis and Timmis 2017, and above).

#### 3.9.2 Health Geography and Spatial Accessibility: Location and Travel Impedance to Healthcare Facilities—Joining Up Healthcare Facilities and Transportation

Not only have healthcare systems evolved organically, but so have their spatial locations, and hence physical access, as have the settlements—villages/town and cities—in which they are embedded. For example, a primary health centre may have evolved from a small doctor's surgery which started at one side of town where a doctor happened to buy/rent a dwelling, whereas over time town expansion may have occurred at the other side of the centre, perhaps with subsequent evolution of public transport networks that complicate access—the issue of health geography and spatial accessibility (Guagliardo 2004). As settlements grow and add healthcare facilities, there may or may not be strategic planning of either their location or public transport networks to minimise access impedance. According to Wallace et al. (2005) ‘about 3.6 million Americans do not obtain medical care because of a lack of transportation in a given year. On average, they are disproportionately female, poorer and older’. Health geography and spatial accessibility is a significant cause of failure to access healthcare services and inequality.

Healthcare needs largely increase with age, some of which are associated with impaired mobility, both walking and driving and cognition (Ferrucci et al. 2016). Superimposed on any underlying mobility issue is the fact that a need to access healthcare is often associated with feeling unwell, which can amplify the problem of accessing needed services. As the elderly age, they increasingly live alone and lack partners, family, and friends who would otherwise facilitate mobility. There is a serious disconnect between patient needs and spatial access to healthcare facilities in some locations.

The same is true of other, often highly dispersed healthcare facilities, such as dental surgeries and physiotherapy and psychotherapy consultation facilities which have created for patients a healthcare centre accessibility challenge in many locations, either in terms of travel distance or public transport connectivity. This fragmentation imposes a significant logistical burden on patients, exacerbating health inequalities and acting as a barrier to the integrated, preventative care model this editorial advocates. There is a fundamental need for integration of evolution of healthcare, town planning and public transportation networks. This requires a paradigm shift: treating equitable physical access as a core parameter of healthcare system performance, governed by policy. This paradigm must explicitly recognise that strategic, transport‐oriented siting is not merely a convenience but a foundational public health and climate mitigation strategy. By significantly reducing the reliance on private vehicles for healthcare access, it directly reduces the sector's substantial carbon footprint and lowers local air pollution—key drivers of respiratory and cardiovascular disease. This integration of health, urban planning, and environmental goals is the definitive operationalisation of the ‘Health in All Policies’ approach. Solutions include legislating ‘Health Accessibility Impact Assessments’ for new facilities, mandating integration between health, planning, and transport authorities and prioritising investment in active travel infrastructure (walkability, bikeability) around care venues as a foundational public health intervention.

An obligatory requirement of DIY Centres is that they are (a) local, that is, community facilities within reach of most people and (b) well‐networked with public transport (Timmis and Timmis 2017). This standard of equitable, community‐centric accessibility must become the benchmark for all new primary care infrastructure. But this needs to extend to traditional healthcare services though solutions based upon systems considerations that integrate town planning, public transportation and healthcare facility access—an elemental aspect of Health in All Policies—through the spatial grouping of diverse healthcare services in ‘one‐stop’ health centres/malls.

The shift from physical to online shopping has resulted in much unused retail space in towns and cities. This change in social behaviour has created a huge opportunity for healthcare systems and town planners to reconfigure healthcare service provider locations and transport networks for optimal accessibility, synergy and sustainability. Capital investments for renovation‐refurbishment‐updating of individual ageing healthcare facilities should consider strategic options for the long term evolution of joined up health infrastructure.

#### 3.9.3 Creation of ‘One‐Stop’ Health Centres/Malls: Joining Up Healthcare Facilities and Their Accessibility

For larger towns, we envisage a pivotal solution to be the creation of new multifunctional—‘one‐stop’—health centres that incorporate classical primary health centres, small outpatient specialist surgeries, DIY facilities, dental surgeries, opticians, audiology centres, physiotherapy and psychotherapy consultation centres, etc., strategically sited for easy access by public transport and active travel networks, existing route‐networks being modified and new ones developed where needed. To move from vision to implementation, governance and funding models such as public‐private partnerships or development led by integrated health and planning authorities must be explored. A pragmatic strategy includes both new builds and the adaptive reuse of existing community assets (e.g., underused retail or administrative space), ensuring solutions are frugal and context specific. Such well‐connected one‐stop‐centres would not only go a long way to solving the problem of healthcare facility fragmentation and physical accessibility, but provide a range of other benefits, such as

- prompt access to other services dictated by an initial diagnosis (see also Muchipa 2023), thereby reducing waiting times and costs, and creating faster, more efficient referral‐diagnosis‐therapy pathways with the improved outcomes this engenders (Hasty 1995; Falces et al. 2008; Tang and Lee 2009; Friedemann Smith et al. 2019),
- economies and advantages of scale, including shared administrative functions and effective use of diagnostic resources
- increased provision of services. As significant attractors for outpatients and outpatient healthcare, they would tend over time to incorporate independent professional diagnostic services, such as exist in some countries like Spain and France, and sophisticated diagnostic‐therapy services such as imaging by MRI and X‐ray, involving stand‐alone machines and technologies,
- replication of services, which would provide increased choice, selection for best services, and, importantly, system resilience
- attraction of medical supplies providers,
- ripple effects that include new opportunities for enterprises and employment. As patient traffic increases, demands for other services, like orthopaedic supplies, and non‐medical services typical of shopping centres/malls, like those providing food and drinks, childcare, health food stores, gyms and sport facilities, etc., would develop and engender new economic development. Critically, these centres operationalise a ‘Health in All Policies’ approach. They become the physical nexus for preventive care and public health education, hosting vaccination drives, nutritional workshops, and health literacy programs, thereby directly addressing the causes of system strain outlined above
- Environmental sustainability by design: The strategic siting at public transport nexuses and the consolidation of services inherently reduce the carbon footprint of patient travel and logistics. Furthermore, these hubs present a critical opportunity to lead by example in the built environment, utilising adaptive reuse of existing structures and employing sustainable construction principles, low‐carbon materials, and energy‐efficient systems for new builds, directly linking urban regeneration to public health resilience,
- sustainability: good public transport links would encourage less use of private cars (lower transportation carbon footprint) and need less parking space (more effective use of land), positioning these hubs as part of climate‐resilient community infrastructure,
- improved logistical sustainability: delivery of materials to one location rather than many different locations will reduce logistical carbon footprints.

#### 3.9.4 Improving Communication and Comprehension Between Healthcare Professionals and Patients

Human migrations over recent decades and especially now have created nations and communities that are more multicultural and introduced new languages into daily communication. Children rapidly learn new languages, in part because they receive their education in, and make friends speaking, national languages, whereas adults tend to learn more slowly, which may present challenges of communication. Poor communication between patients and healthcare professionals can be particularly problematic because failure to properly understand clinical pictures can lead to misdiagnosis and failure to understand therapy regimes can lead to a lack of compliance. e‐Clinician Centres have the advantage of scale that not only provides a range of specialist expertises but also the ability to communicate in the range of languages used in their country. This communication‐comprehension joining up will represent a significant step towards reducing inequalities engendered by language issues.

### 3.10 Sustainability as a Pillar of Healthcare Resilience

Long term wellbeing, survival and perpetuation of *Homo sapiens* require that policies and practices at all levels of society become sustainable. The integration of sustainability considerations in relevant policy discussions and development is foundational and existential. The proposed recasting of primary healthcare is not merely a defensive measure against healthcare system vulnerabilities and inequalities, but also an active strategy for sustainability, climate mitigation and environmental health, creating a virtuous cycle that reinforces systemic resilience. This is a moral imperative: a healthcare system that fails to mitigate its own environmental impact is ultimately harming the planetary and community health it is sworn to protect, violating its fundamental duty of care. By design, the model we propose generates profound sustainability co‐benefits that translate the abstract goal of ‘Health in All Policies’ into concrete, measurable outcomes.

#### 3.10.1 The Co‐Benefits of a Low‐Carbon, Accessible Health System

A primary source of these benefits is the radical improvement in logistical efficiency and patient‐centric design. The shift from suboptimally located healthcare facilities with poor connectivity to a distributed network of local DIY Digital Medical Centres and strategically sited One‐Stop Health Centres (see below), all mandated to be linked by frequent, affordable public transport, would dramatically reduce the reliance on private vehicle travel for routine care. This could potentially avoid millions of car trips annually in larger nations, directly translating into significant reductions in metric tons of CO_2_ emissions. This aligns the healthcare system with net‐zero goals and reduces its indirect environmental burden.

#### 3.10.2 Systemic Co‐Benefits Beyond Carbon

Reductions in private car use will reduce traffic congestion and yield immediate public health and economic dividends: lower emissions of particulate matter (PM2.5) and nitrogen oxides (NOx) improve air quality, directly addressing risk factors for childhood asthma, cardiovascular disease and other non‐communicable diseases. Furthermore, it saves time and fuel costs for citizens and reduces public expenditure on road maintenance and expansion. This exemplifies the ‘One Health’ principle, where an intervention in healthcare delivery positively ripples through the environmental and social determinants of health.

#### 3.10.3 Sustainable and Adaptive Infrastructure

The physical implementation of this model champions sustainable design. It prioritises the adaptive reuse of existing community assets, such as declining retail spaces, for healthcare hubs, contributing to a circular economy. New builds, including low‐cost hospitals and recovery facilities, can be designed from first principles for energy efficiency, utilising sustainable materials and construction principles. This approach both minimises the embedded carbon of the healthcare estate and creates infrastructure that is inherently more resilient to climate extremes and adaptable to future needs.

Embedding sustainability into the architecture of healthcare delivery significantly reinforces the core argument: this reconstitution represents a systemic improvement. It moves beyond clinical and logistical gains to generate foundational co‐benefits for planetary and public health, ensuring the system is not only resilient to external shocks but also a proactive contributor to a healthier, more sustainable future.

### 3.11 The Pivotal Role of Microbes in Health, Healthcare and Healthcare Recasting

#### 3.11.1 Microbes Are Life

Microbes are the origin of life: ancient microbes were our ancestors, and we have evolved as human:microbe composites, with microbially‐derived mitochondria inside most of our body cells providing us with energy, and multitudes of microbes on our body surfaces providing all manner of other services. Microbes are characterised by an incredible phylogenetic, physiological and metabolic diversity that has enabled them to thrive under a wide range of environmental conditions found on the surface and in the subsurface of the planet. The habitats they occupy/create define the biosphere. In these diverse habitats they access carbon and energy for growth from a wide variety of substances, a global dynamic planetary metabolism that continuously cycles materials, including biological wastes, industrial products and environmental pollutants, disassembling biological, geological and synthetic materials into their component parts and making them available for the sustenance of existing organisms and creation of new life. In degrading industrial pollutants, microbes protect us from self‐inflicted chemical intoxication. Many microbes are also autotrophic—they grow and reproduce using carbon dioxide and either chemical or solar energy—and hence, like plants, are primary producers at the base of diverse food chains/webs, sustaining life on Planet Earth, including the lives of *Homo sapiens* . Microbial activities and biomass production vitalise the life we know. Their small size, relative simplicity, and fast reproduction rates make microbes preferred biological systems of choice for studying basic life processes. Our understanding of health and disease, and what we can do to influence them, is due in large part to the study of microbes (Timmis, Baquero, et al. 2025).

#### 3.11.2 Microbiomes Are Intimate Metabolic Partners in the Cycle of Life

Essentially all higher organisms are covered in microbes—their microbiomes—which provide a huge range of vital services and have a major influence on organismal health. Microbiomes and their services are essential to life: so‐called germ‐free organisms have poor health and short lives. Microbiomes play major health‐promoting roles in human health. Microbiomes are a vital integral part of all higher organisms which are therefore *metaorganisms, holobionts, unified superorganisms*. Microbiomes are acquired by humans at birth, accompany them throughout life, and initiate the recycling process when they expire (Armstrong and Timmis, submitted). The microbiome contributes about half of all the cells of the human metaorganism. Crucially, it also contributes more than 99% of its genes, the expression of which translates into the synthesis of a vast cocktail of metabolites that have profound consequences for human physiology and health, consequences that offer a range of possibilities for healthcare interventions.

#### 3.11.3 Microbiomes Make Up the Microbiosphere, a Microbial Metaconnectome Globally Linking All Organisms of the Biosphere

Microbiomes are not static but highly dynamic, continually exchanging members with the environment and varying with inter alia environmental context, nutrition, ageing, lifestyle, medication. Human microbiomes acquire new members via the gastrointestinal tract (gut) from food and drink, via the airways from air, via the skin from contacts with other humans, companion animals, plants, soil, inanimate surfaces, etc., and deliver existing members to the environment via the gut, lungs and skin. The microbial world—the microbiosphere—is a continuum of interconnected microbiomes of organisms and environmental habitats, a global biological ‘connectome’ linking all forms of life with one another and with the planetary media: air, water and soil/sediments (Timmis, Baquero, et al. 2025).

The transmission of infectious diseases is a facet of the connectome because it links pathogens from reservoirs to hosts. Examples include *Vibrio cholerae* thriving in crustaceans that are consumed without cooking by humans, transmission of respiratory infectious agents such as influenza and SARS‐CoV‐2 via air containing droplets originating from infected airways, fungal infections by air and soil, antimicrobial resistant opportunistic pathogens via surfaces in hospitals, *Plasmodium* developing in mosquitoes and *Borrelia* replicating in ticks that need a blood meal for their reproductive cycles, *Salmonella* and Hepatitis A virus that contaminate untreated water, *Legionella* growing in water and air conditioning systems and in amoebae, *Mycobacterium avium* growing in hot tubs and spas, and direct contact infections like sexually‐transmitted infections (STIs).

The microbiosphere connectome not only provides routes of transmission of infectious diseases but also opportunities for transmission interruption (barriers). These include the use of probiotics for surfaces—safe and robust microbes that outcompete pathogens and can be applied to contact surfaces, such as those in hospitals (D'Accolti et al. 2024) or in places characterised by high levels of human traffic, such as public transport stations, that carry multi‐resistant opportunistic pathogens (D'Accolti et al. 2023), face masks to restrict the transmission of respiratory pathogens, wastewater treatment to destroy faecal pathogens, various food sterilisation processes and condoms to prevent contact transmission of STIs. As understanding of the microbiosphere and the transmission processes it mediates increases, new strategies to prevent transmission will become apparent.

#### 3.11.4 Microbiomes Are Our Second, Outer Skin, the First Point of Contact With the Environment

Our microbiomes cover all our external surfaces and are thus considered to constitute our second, outer skin. They are the first point of contact with the environment and substances delivered to the human body. These include skin contacts and materials transported into airways during breathing. Most important, however, is the gut microbiota which is the first contact with food, alcoholic and non‐alcoholic drinks, prescription drugs, non‐prescription substances, environmental toxins and everything else taken in orally. This contact is highly metabolic resulting in the conversion of many ingredients into diverse metabolites, some of which have positive effects on health, others of which have negative effects, and yet others which have no effect. For example, the gut microbiota may degrade and thereby protect from toxins such as agrochemical residues on plant foods, but in other cases may convert them to more toxic metabolites. Similarly, gut microbes may metabolise medicines, creating more active or inactive metabolites, thereby affecting drug efficacy, toxicity and pharmacokinetics. Understanding the metabolic and resulting physiological consequences of microbiome:environmental substance interactions will offer new insights that translate into new opportunities for precision health interventions.

#### 3.11.5 Microbiomes Pervasively Influence the Functioning of Body Control Systems

All activities of the human body are steered and controlled by a hierarchy of regulatory systems that operate at levels ranging from the individual gene to global systems of whole‐body control. The latter includes the nervous system operating through electrical impulses and neurotransmitters, the endocrine system operating through hormones, and the immune‐inflammatory system operating through cytokines which constitutes the frontline response of the body to infections, injuries and other forms of disease. These global regulatory systems are exquisitely integrated and interdependent such that the extraordinarily complex ecophysiology of healthy humans runs optimally most of the time (O'Riordan et al. 2025), While there is a reasonably good understanding of how the human components of these systems function, there is considerable uncertainty about the impact and role of the microbiome.

Associated with the gut is a component of the nervous system—the enteric nervous system (or second brain)—which communicates with the brain via the vagus nerve. Gut microbe‐created metabolites identical to or resembling neurotransmitters can directly influence the enteric nervous system and thence the brain: the microbiota‐gut‐brain axis (Cryan et al. 2019; Cryan and Mazmanian 2022). Other metabolites have hormonal activity, which is why the gut microbiome is regarded as the second endocrine system (Clarke et al. 2014). And yet others, for example short chain fatty acids, regulate the immune system (Rooks and Garrett 2016; Zheng et al. 2020; Takeuchi et al. 2024). Such microbial metabolites exhibiting neurotransmitter, neuroendocrine and neuroimmune activities profoundly influence body control systems, human physiology and neurological processes.

Superimposed on the complex interactions of human hormones and microbiome originating hormones/hormone‐like metabolites, are pollutants that, while not being toxic in the sense of killing cells at the concentrations experienced by humans, can interfere with hormonal controls—the so‐called endocrine disrupting chemicals (ECDs)—and hence exert significant effects on human physiology. Estrogenic endocrine disruptors cause reproductive disorders in both females and males and thereby reduce fertility (Kiyama and Wada‐Kiyama 2015; Cripps et al. 2024). Currently the metabolic fate of ECDs during exposure to the gut microbiome is poorly understood but falling fertility rates (Bhattacharjee et al. 2024) are consistent with estrogenic disrupting activities being maintained or perhaps even potentiated in the gut.

#### 3.11.6 The Gut Microbiota Chemical Factory: A Vast, Poorly Understood Regulator of Health and Target for Interventions to Prevent and Treat Disease

Given that the major control systems of the body are highly integrated, impacts by molecules produced by gut microbes can have multiple and/or profound effects on physiology and health, especially mental health and perhaps in polymorbid patients. In addition to inherent genetic, lifestyle and environmental differences between individuals, different people have different microbiome compositions, so there is another layer of patient variability to be addressed by precision medicine. Perturbation of the composition of gut microbiota, for example by a change in diet, intake of oral medicaments, consumption of recreational drugs, exposure to pollutants including EDCs, etc., entrains a change in the metabolites produced in the gut and the consequences they have for health. All of this implies that there is a huge landscape of potential microbiota:host interactions that can be explored for targeting by diet, pre−/probiotics, drugs, microbiota structuring, etc., to effect desired outcomes.

### 3.12 Mental Health and Well‐Being: A Core Dimension of Universal Healthcare Requiring Dignity, Access and Integration

‘Mental, neurological and substance use conditions account for 14% of the burden of disease. Between 75% and 90% of people with these conditions do not receive the treatment they need even though effective treatment is available’ (World Health Organisation 2008; Kohn et al. 2004). This gap constitutes a catastrophic failure to uphold the human right to health with dignity. While a deficit of psychiatrists is certainly a part of the problem, and treatment costs are another, the unpredictable nature and especially timing of onset of symptoms that need professional help at any time of the day or night is a characteristic aspect of many mental disorders. This need is partially satisfied by some telemedicine and online support services, such as Your Hope Line (https://yourhopeline.com), Minds Foundation (https://www.mindsfoundation.org) and the Samaritans (https://www.samaritans.org). However, finding such services during a crisis in the middle of the night may be challenging for some, particularly in the context of problems of availability and accessibility of general healthcare. While clinically‐approved Apps can provide appropriate support in certain circumstances, having mental health professionals available online 24 h per day, 7 days per week within a single primary healthcare digital ecosystem, providing services in all relevant languages, would create a comprehensive support system that would undoubtedly improve care and outcomes of neuropsychiatric disorders and decrease patient anxiety caused by healthcare access uncertainty.

Physical and mental disease have traditionally been two separate branches of medicine. However, as discussed above, the gut microbiota plays diverse roles in both physical and mental health and disease, thereby directly linking the two. The composition and functioning of the gut microbiota are themselves influenced by nutrition and lifestyle. Research on how the gut microbiota influences mental processes, in particular increased focus on context, mechanism and causality, will provide the resolution required to increase our understanding of mental processes and health in general, and reveal new dimensions of potential microbiome‐centric treatment options for prevention, diagnosis and treatment of mental health disorders. These will include procedures to modulate gut microbiome composition and function involving pre‐, pro‐ and synbiotics/neutraceuticals and microbiome transplantation therapy, which has already been remarkably successful in treating recurrent/persistent *Clostridioides difficile* infections. The proposed digital ecosystem—with 24 h per day, 7 days per week access to the e‐clinician centre, AI‐supported triage in DIY Centres, and anonymised population‐level monitoring by the NCIC—provides the foundational architecture to finally close this gap, delivering dignified, accessible and integrated mental healthcare as a standard component of universal coverage.

This holistic view must also address important social determinants of health like loneliness—a source of significant suffering that affects all ages but is particularly acute among the elderly. It is not a classified disease, but its impact on mental and physical health can be severe and lifespan shortening. A reconstituted, connected healthcare system, with its community‐based DIY Centres, health education services and digital check‐ins, could provide vital points of regular social contact and monitored well‐being, combating the isolation that current, transactional health models often ignore.

### 3.13 Unlocking Resources and Capacity: Incentivising Development of Frugal Sustainable Microbial Technologies to Increase Healthcare Accessibility and Reduce Inequalities

#### 3.13.1 Microbial Diversity—Applications in a Wide Range of Sectors

The vast diversity of microbes (one estimate suggests that there are 1 trillion species: Locey and Lennon 2016; for comparison, estimates of the number of species of higher organisms are typically in the millions), their physiologies and metabolisms provide an almost unlimited number of diverse activities and products that can be exploited in a wide spectrum of biotechnological applications that directly or indirectly promote human health.

The ease of study of relevant microbes permits rapid characterisation and redesigning/repurposing of interesting properties by genetic‐genomic approaches, and variants with desired traits can be conveniently sought by simple large‐scale assays/detection systems and robotics. Their small size also lends them to whole organism applications, miniaturisation, lab‐on‐a‐chip technologies, and especially to powerful synthetic microbiology approaches. Microbes provide products and activities and serve as inexpensive and sustainable ‘cell factories’ to produce microbial and non‐microbial products. The field of microbial technology is characterised by exceptional innovation. Microbial technologies are special in that they are considerably more sustainable than traditional technologies: they mostly operate at low temperatures, typically 20°C–30°C, and hence are low energy‐requiring and low cost, mostly do not involve the use of toxic chemicals and mostly are not pollutant emitters. Furthermore, their production often relies on simpler, localised bioprocesses, reducing dependence on complex, energy‐intensive global supply chains characteristic of many synthetic pharmaceuticals and high‐tech equipment. They are ideal processes for healthcare innovation and especially for advancing health equality in low resource settings.

#### 3.13.2 Applications of Microbial Technologies in the Service of Human Health

Microbes have delivered vitally important applications in diverse sectors such as nutrition (bread, cheeses, alcoholic beverages, whole cell protein, fermented foods, etc.), pathogen control through wastewater treatment and drinking water production, pollution control, bioenergy production (all of which are health‐relevant), but their most significant impacts have been directly in the health sector which has experienced extraordinarily rapid progress. Many prophylactics and therapeutic drugs are based on microbes, microbial natural products, or are manufactured in microbial cell factories and thus constitute microbial technologies. Microbes have delivered/inspired a wide range of pharmaceuticals, including antibiotics that have saved untold lives and vaccines that have protected billions from life‐threatening infections, witness the COVID‐19 vaccines, and powerful diagnostics.

More recent developments include

- diagnostics, such as polymerase chain reaction (PCR), loop‐mediated isothermal amplification (LAMP) and genomics procedures and biosensors, for centralised and point of care/environmental diagnostics and environmental surveillance, including pathogen monitoring in wastewater (Girón‐Guzmán et al. 2024),
- synthetic RNA vaccines (Sevilla et al. 2024)
- ‘microbots’ for diagnosis and treatment of disease (Jin et al. 2025)
- live weaponised cancer‐homing microbes or microbial vesicles for cancer treatment (Brahmbhatt and MacDiarmid 2021)
- new biocompatible materials for clinical applications, like wound dressings (Rivero‐Buceta et al. 2020),
- probiotics (including probiotics of surfaces of the built environment to reduce transmission of antimicrobial resistant pathogens; D'Accolti et al. 2023, 2024),
- microbiota remodelling agents, including faecal material transplants and simplified versions of these (Bratkovič et al. 2024),
- recovery of medical resources, such as contrast agents, that are expensive and/or available in limiting quantities (Good et al. 2024)

The innovation potential of microbial products and processes is exceptional. This is the *Age of Microbial Technology* (Timmis and Hallsworth 2024).

#### 3.13.3 The Problem of Antimicrobial Resistance

The inexorable rise in antimicrobial resistant (AMR) microbes has precipitated a major health crisis with AMR pathogens predicted to become the leading cause of morbidity by 2050, with 10 m deaths annually and a cumulative loss of economic output of $100 trillion (O'Neill 2016). There is an urgent need for new and effective therapies for potentially lethal infections caused by AMR pathogens. Promising options currently being explored are mostly microbial in nature and include therapeutic vaccines, microbially‐produced antibodies (nanobodies), parasites of pathogens, including viruses and bacterivorous bacteria, like *Bdellovibrio*, and microbiome transplants (e.g., for *C. difficile* colitis).

Microbes are all pervasive in human health and disease and offer a multitude of possibilities for interventions in healthcare. Consideration of microbial technologies must therefore be central in strategic discussions about resets.

### 3.14 Unlocking Resources and Capacity: The Central Importance of Health Education

We have indicated above the importance of basic healthcare education in school to promote awareness of the importance of healthy lifestyles and behaviour, and to counter any unhealthy lifestyle culture in the community. However, healthcare education at school is even more important for personal engagement in healthcare because an understanding of what might be going on in the body, why a certain type of diagnosis is appropriate, what it tells the clinician, what a diagnosis means in terms of treatment, why treatment schedules must be strictly observed, what factors influence disease transmission and how they can be influenced, is key to patient agency. Moreover, it opens a window to healthcare information and enables comparisons, benchmarking and understanding of quality healthcare and best practice. Crucially, it is needed to counter poor and inaccurate advice available on the world wide web because having a basic evidence‐based knowledge and understanding of health and disease will not only obviate some needs for information but will also make information searches more focused, objective and critical. It is therefore vital to begin to design, create and implement a citizen's healthcare education curriculum.

Exhortations to live healthily have only had limited success. While the reasons are diverse, habits die hard and peer pressure is a powerful influence, as can be seen by alcohol consumption and smoking activity at parties. But peer pressure can be positive as well as negative; witness the rise in jogging and exercise in the gym. There is a growing awareness of the importance of exercise for both health and personal appearance, but this comes mostly for those in their 20s and beyond. School children are still susceptible to the development of unhealthy habits, especially through peer pressure, and many have home lives characterised by unhealthy lifestyles that heavily condition the habits of the children. How to extend the influence of healthy lifestyle peer pressure operative on the 20+ generation to the 10+ generation? How to get the 10+ generation invested in their own health?

One way that would undoubtedly have significant long‐term health, and all the related benefits, would be mandatory health education in school. All children, not just those electing to study biology, should learn about their bodies, how they work, how things go wrong, how things can be prevented from going wrong, and what the consequences are of not preventing things going wrong. The mandatory curriculum must include mental health literacy: understanding emotions, stress, the mind–body connection, breaking down stigma and knowing how and when to seek help for oneself or others. Crucially, they must be taught about the central role of their own microbes in the process of health and disease. This should be complemented by information on the multitude of societal costs of ill health. Mandatory health education in school would create a universal understanding of health and disease, and many children would become invested in their own well‐being and in the care of their microbiomes. This might well create a school environment in which peer pressure would be directed more towards healthy pursuits than unhealthy ones, such as smoking/vaping and alcohol and substance abuse, as is the case now in many settings.

Becoming invested in one's own health is thus far largely age‐related: the greater the likelihood of ill‐health, the greater the degree of interest in preventing it. The task is therefore to lower the age of becoming invested in health. Education may facilitate this.

Proposals to change school curricula are often countered by statements that such curricula are already overcharged and teachers have neither time nor energy to adopt new disciplines. While we readily accept that teachers are overworked, and that this situation urgently requires resolution, there is a lot of discussion about the need for curriculum change, that current curricula no longer provide children with the knowledge, understanding and skills needed in the 21st century. Many of these discussions, however, are workforce needs‐centric, as though the health, resilience and societal adequacy of the economy is regulated primarily by available skills. However, as we have discussed, healthcare is a major element of the economy, both in terms of financial costs and productivity (not only days lost through illness and need for carers, but also lost productivity resulting from reduced creativity‐innovation), so creating a society engaged in and committed to elevating health‐reducing disease will have a significant impact on economic performance and resilience. Mandatory education in healthcare and microbiology will be central to achieving this.

Two other aspects of health education should be mentioned. The first is the relatively low cost of rolling out a mandatory curriculum in health education. While such a curriculum would have major health and societal benefits that would also translate into significant economic benefits, unlike somewhat controversial policy decisions about for example reducing carbon emissions, which require both major societal adjustments and huge investments of taxpayer revenues, the basic infrastructure for a curriculum in health education is in place and the importance of it is obvious and thus likely to be welcomed by most of the general public.

Another important issue is Service Learning, which combines academic learning with community service (Jacoby 1996; Aramburuzabala et al. 2019; Harpine 2024), typically serving the underprivileged, such as those with learning difficulties (Harpine 2024), or in shelters for the homeless (Reeb et al. 2024; Valderrama et al. 2025). As a result of its broad relevance to humans and society, microbiology is an ideal subject for service learning (Webb 2017), in particular health‐relevant aspects, such as sexually‐transmitted infections, antibiotic resistance, and so forth, that speak directly to situations faced by target community members (Valderrama et al. 2025). Service learning has the aim and potential to increase the dignity of those taught and help them acquire information pertinent to their lives, and to provide the students involved with greater appreciation of societal inequities and humanitarian needs. Service learning related to health and disease contributes to health education in marginalised groups and a reduction of inequalities in healthcare accessibility.

Service learning, especially in the context of health education, should be substantively expanded and become an integral component of educational efforts to reduce inequality and improve access to healthcare. It must be incentivised by a broad group of actors, including university leadership, educational authorities, politicians, social service leaders and international organisations.

### 3.15 Equitable Global Financing/Health Insurance to Achieve Better Healthcare Access

Most HICs have universal health financing systems in place that allow for access to basic healthcare services, notwithstanding various problems and inequities characteristic of each type of system. However, many LMICs simply do not have adequate financing systems that provide any degree of universal cover. According to one estimate, only 8% of people in low‐income countries are covered by health insurance (Hooley et al. 2022). This constitutes one of the most serious causes of social inequity. HICs are morally and ethically obligated to take on more global responsibility for universal health coverage and overall reduction of risk health risks, such as global pandemics, and must therefore engage in development of supranational financial instruments to fund global equitable healthcare. To address this, a global, risk‐adjusted healthcare fund to cover the costs of diagnostics and prophylactic and therapeutic interventions is urgently needed. This fund's allocation decisions must adhere to the ‘Accountability for Reasonableness’ framework, ensuring that rationales for limiting care are transparent and justifiable to ‘fair‐minded’ individuals affected by those decisions (World Health Organisation 2015).

### 3.16 Unlocking Resources and Capacity: The Importance of Strategic Investment in Infrastructure Innovation in Low Resource Settings

Societal inequalities tend to be multiple and reinforcing. Poverty often implies unhealthy living conditions, poor nutrition, increased probability of infectious disease due to poor access to clean water, inadequate awareness of important hygiene measures because of limited access to education, and so forth, all of which can amplify the risk of disease in situations in which access to healthcare may be seriously limiting. While the simultaneous solution of multiple issues can be challenging, targeting investment to galvanise the greatest multifactorial long‐term benefit, through systems analysis, is urgently needed. For example, investing in

- low‐cost primary healthcare facilities, including DIY Healthcare Centres, will increase healthcare access, reduce the incidence and severity of disease and its collateral damage of reduced productivity and thereby mitigate poverty;
- increasing the range of frugal healthcare materials, instruments and technologies will lower costs and increase availability.
- local production of locally needed diagnostics and therapeutics can not only reduce poverty but also rejuvenate and revitalise disadvantaged communities and their economies;
- wastewater treatment and drinking water supply will reduce infections and associated collateral issues;
- education of disadvantaged communities can enable the inherent talent potential of individuals to be realised and raise the value of the workforce of such communities;
- health education of such communities can help them achieve better states of health and some to become health professionals themselves;
- better housing for those in informal settlements will help diminish physical, mental and conflict issues associated with overcrowding.

In an earlier discourse (Timmis et al. 2014), we made the link between such investment and investment for economic recovery and growth, because it grows a vital knowledge‐based bioindustry/bioeconomy. Moreover, for HICs to create such bioindustries in LMICs will not only produce needed new prophylactics and therapies—crucially new prophylactics and therapies relevant to local diseases—but also seed bioindustries that can serve as enterprise nuclei which may, with appropriate support and nurturing, develop into hubs of economic activity, thereby countering poverty and contributing to social equity and local community wellbeing.

## 4 Concluding Remarks

Crisis to Covenant‐A Roadmap for the Fundamental Duty of CareThe central paradox of 21st‐century healthcare is that our capacity to diagnose and treat disease has never been greater, yet our systems are failing to deliver this potential to more than half of the world's population. This is not merely a technical or financial shortfall; it is a systemic and ethical failure. As this editorial has argued, overcoming it requires more than incremental adjustments. It demands a fundamental change in mindset of all healthcare ecosystem actors, including the general public, of the type and scale articulated by Merz et al. (2023) in relation to ecological overshoot, and a fundamental recasting of healthcare purpose, priorities, governance and innovation to achieve universal healthcare and align with the principle of best health outcome practice.

The point of departure for this reset must be the explicit recognition of a non‐delegable public duty. Governments hold the fundamental responsibility to ensure universal, quality healthcare for their constituents. While the *provision* of services may be delegated, this *obligation* and ultimate accountability cannot be abdicated. Therefore, any delegation—especially to profit‐driven private entities—must be matched by stringent, enforceable frameworks for quality, equity and access. A system's failure to meet these standards constitutes a dereliction of duty. This principle must become the bedrock of all healthcare policy, transforming ‘healthcare as a human right’ from a declarative slogan into a measurable, enforceable covenant. This covenant must be grounded in enduring principles of bioethics, including justice, beneficence, nonmaleficence, and respect for autonomy (Beauchamp and Childress 2019). Its ultimate purpose is to dismantle the structural violence embedded in current health systems—the avoidable harm caused by policies, economic arrangements and power asymmetries that systemically deny dignity and health to some groups of society, in particular those currently marginalised (Galtung 2013).

With this duty as our foundation, the path forward is a strategic integration of three synergistic forces.

### 4.1 The Digital and Patient‐Agency Revolution

We must architect systems for ‘Networked Agency with a Safety Net.’ This means actively empowering patients through DIY Digital Medical Centres and Home Clinics, underpinned by National Clinical Informatics Centres. This digital ecosystem does not abandon patients to self‐care but uses AI and integrated data to enable safe, guided autonomy while ensuring continuous professional oversight and preserving the collective safety net. It flips the script from passive recipients to active participants, unlocking vast primary healthcare system capacity.

### 4.2 The Microbial Technology Frontier

Our understanding of the microbiome and microbial technologies represents a paradigm shift in medicine. From diagnostics and vaccines to mental health and sustainable production, microbial innovations offer uniquely frugal, scalable and sustainable tools. Prioritising this frontier is key to developing affordable, context‐appropriate solutions for both HICs and LMICs, directly attacking inequalities.

### 4.3 The Holistic, Preventive Re‐orientation

**FIGURE 1:**
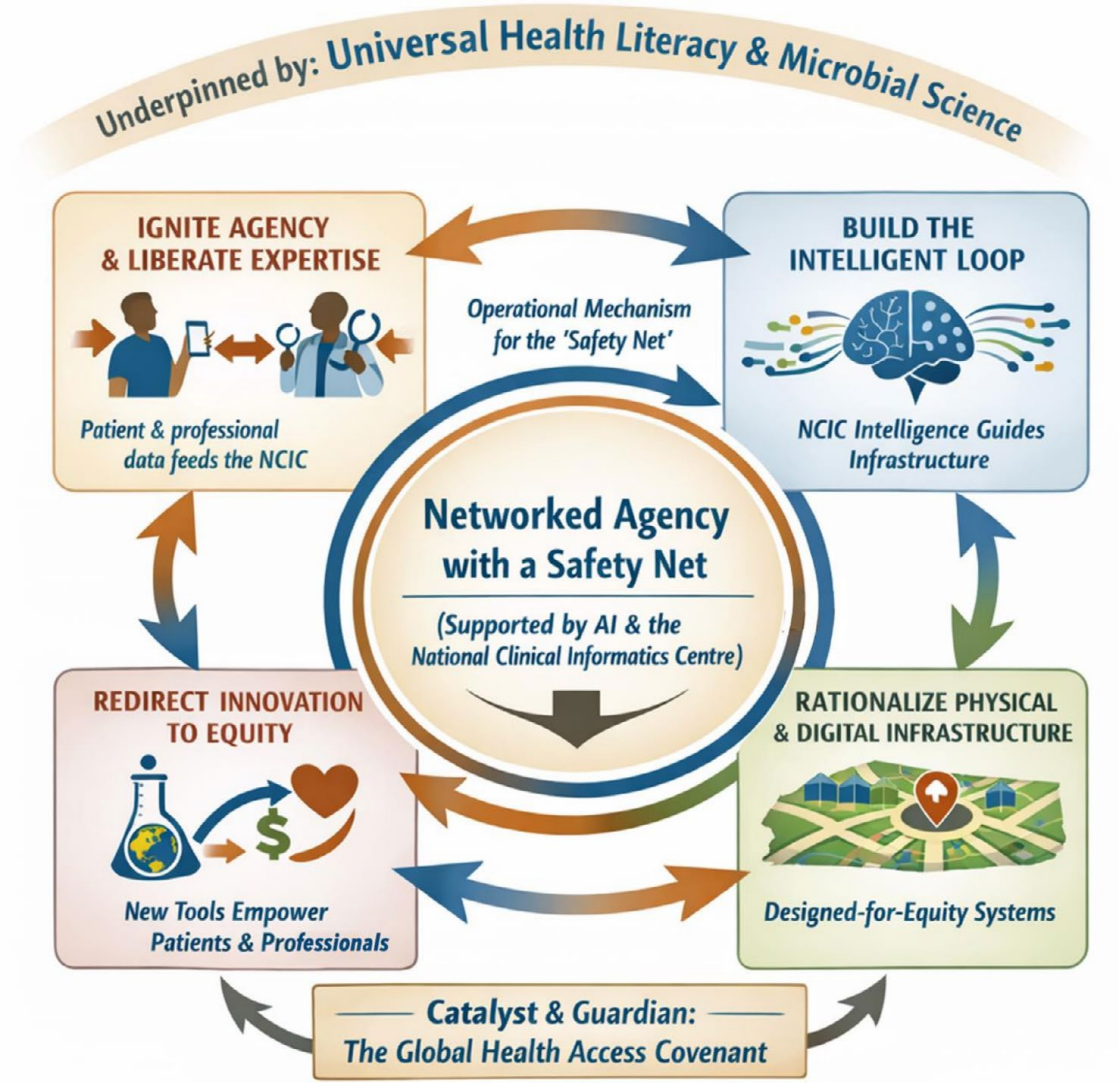
The virtuous cycle of healthcare system reconstitution. This diagram illustrates a self‐reinforcing framework for rebuilding equitable, resilient health systems through *networked agency supported by a national safety net*. Four interconnected domains—(1) igniting patient and professional agency, (2) building an intelligent clinical informatics loop, (3) rationalising physical and digital infrastructure and (4) redirecting innovation towards equity—form a continuous cycle of enablement and feedback. At the centre, artificial intelligence‐supported national clinical informatics provides the operational mechanism that coordinates individual agency with system‐level protection. The cycle is underpinned by universal health literacy and microbial science, and catalysed by a Global Healthcare Access Covenant that initiates and safeguards systemic transformation. NCIC, National Clinical Informatics Centre.

A resilient system cannot chase disease endlessly. It must be designed to prevent it. This requires integrating the ‘Health in All Policies’ approach—from urban planning and education to climate resilience—and making comprehensive primary, preventive and mental healthcare the undisputed core of service delivery (Figure 1).

Most healthcare systems are chronically underfunded. However, funding is only one element of the solution equation, because funding use is often suboptimal and efficiencies can be improved (OECD 2017). Moreover, there exists significant system capacity potential that can be unlocked through the recasting of healthcare systems proposed here. This includes:

- actors (healthcare professionals and patients, through patient education, empowerment and engagement in their own health),
- productivity (through accelerating uptake and centralising digital technologies),
- hospital capacity (through creation of new low‐cost facilities)
- primary healthcare capacity (through advancing frugal technologies)
- healthcare accessibility (through linking facilities with efficient public transport networks)
- reduction/constraint of demands for services (through greater efforts to improve population health)

Importantly, significantly improving population health, preventing disease and shortening pathways of disease diagnosis, treatment and recovery are not only key to wellbeing but also to achievement and productivity of the individual and the population. The additional system capacities unlocked by the measures proposed here, and the resulting increase in population prosperity and productivity will in turn raise tax revenues available for public services, some of which can be used to further improve healthcare services: the virtuous cycle of Darzi (2024).

## 5 A Strategic Roadmap for Action

Our recommendations are not a menu of options, but the interconnected pillars of a new strategic architecture designed to fulfill the fundamental duty of care.

### 5.1 Establish the Governance Foundation: The Duty of Care

- Anchor policy in the principle of non‐delegable public responsibility. Enshrine in policy frameworks that government accountability for universal, quality outcomes is absolute, regardless of delivery models.
- Mandate ‘Networked Agency with a Safety Net’ as the design standard for all digital health initiatives, ensuring patient empowerment is coupled with robust, AI‐enabled clinical oversight and systemic safeguards.

### 5.2 Re‐architect the Delivery System for Access and Resilience

- Launch a national rollout of low‐cost access nodes: Create the infrastructure for patient agency by investing in networks of DIY Digital Medical Centres and supporting Home Clinic ecosystems, linked to a National Clinical Informatics Centre.
- De‐silo care through integrated ‘health hubs’: Develop one‐stop health centres that co‐locate primary, specialist, mental and social care, strategically sited with public transport links, to shatter physical and bureaucratic barriers to access.
- Create tiered facility portfolios: Systematically develop low‐cost hospital and recovery facilities to free high‐cost, high‐acuity resources for those who truly need them, increasing overall system surge capacity.

### 5.3 Reorient Innovation Towards Equity and Sustainability

- Direct R&D and investment through the lens of frugality and equity. Establish public‐private incentive funds specifically for: (a) frugal diagnostics and mobile technologies, (b) microbiome‐based interventions for mental and physical health and (c) vaccines and treatments for neglected diseases.
- Incentivise and mandate sustainable design and operation: Establish guidelines and funding mechanisms to ensure all new and retrofitted healthcare infrastructure (DIY Centres, One‐Stop Hubs, low‐cost hospitals) are sited for optimal public transport access, built to high energy efficiency standards, and operate on circular economy principles. This will reduce the health sector's environmental footprint while improving community health.
- Decentralise and democratise medical innovation: Build R&D and bio‐manufacturing capacity in LMICs, ensuring the global research agenda is driven by global burden‐of‐disease, not market size alone.
- Make microbial technology a cross‐cutting priority in health, environmental and industrial innovation strategies.

### 5.4 Build Human Capital: From Clinicians to Citizens

- Liberate clinical expertise: Conduct and act on audits of clinician time, using digital tools and new professional roles to remove bureaucratic and routine task burdens, redeploying expertise to complex care.
- Launch a universal ‘Citizen's Healthcare’ curriculum: Implement mandatory, multi‐year education in schools on body–mind health, microbiology, nutrition and digital health literacy to create a generation of empowered, health‐literate citizens capable of true partnership in their own care.

### 5.5 Secure Global Solidarity

- Develop new global financing instruments for universal health coverage, including risk‐pooled mechanisms to fund essential services in LMICs and secure global public goods like pandemic preparedness.

This roadmap is ambitious but essential. The barriers—vested interests, inertia, siloed thinking—are formidable. Overcoming them requires framing the challenge as the fulfilment of a fundamental covenant, rather than a technocratic puzzle. The technologies and strategies we outline are not merely innovative; they are the practical, modern means to uphold an ancient duty: to care for one another, to leave no one behind, and to build healthcare systems worthy of the health we have the knowledge to create.

A covenant and roadmap, however compelling, remain aspirational without the systems and structures for their implementation. Translating this vision into reality demands a resurgence of bold, public‐interest leadership committed to equity and sustainability. To operationalise this commitment, we call upon universities, governments, international organisations and philanthropic agencies to establish and robustly resource dedicated institutes for the critical, interdisciplinary work ahead. These must include, but not be limited to, centres focused on: Primary Healthcare Accessibility and Equity, Health‐ and Sustainability‐Centric Integrative Urban Planning, Universal Health Literacy and Education, and Frugal, Sustainable Healthcare Innovation. Such institutions will generate the evidence, train the leaders, design the policies and forge the partnerships necessary to turn this manifesto into measurable progress. The reset begins with this commitment.

## Author Contributions

**Kenneth Timmis:** conceptualization, writing – original draft, writing – review and editing. **Gerard Clarke:** writing – review and editing. **María Francisca Colom:** writing – review and editing. **Zeynep Ceren Karahan:** writing – review and editing. **Rachel Armstrong:** conceptualization, writing – review and editing, visualization.

## Funding

The authors have nothing to report.

## Conflicts of Interest

The authors declare no conflicts of interest.

## Data Availability

The authors have nothing to report.
